# Deep learning model for the prediction of all-cause mortality among long term care people in China: a prospective cohort study

**DOI:** 10.1038/s41598-024-65601-4

**Published:** 2024-06-25

**Authors:** Huai-Cheng Tan, Li-Jun Zeng, Shu-Juan Yang, Li-Sha Hou, Jin-Hui Wu, Xin-Hui Cai, Fei Heng, Xu-Yu Gu, Yue Zhong, Bi-Rong Dong, Qing-Yu Dou

**Affiliations:** 1 Department of Biotherapy, Cancer Center, West China Hospital, Sichuan University, Chengdu, China; 2 Laboratory of Cardiac Structure and Function, Institute of Cardiovascular Diseases, West China Hospital, Sichuan University, Chengdu, China; 3 West China School of Public Health and West China Fourth Hospital, Sichuan University, Chengdu, China; 4 International Institute of Spatial Lifecourse Health (ISLE), Wuhan University, Wuhan, China; 5 National Clinical Research Center for Geriatrics, Center of Gerontology and Geriatrics, West China Hospital, Sichuan University, No. 37 Guoxue Alley, Wuhou District, Chengdu, 610041 China; 6 Department of Mathematics and Statistics, University of North Carolina at Charlotte, Charlotte, NC USA; 7 School of Medicine, Southeast University, Nanjing, China; 8 Department of Cardiology, West China Hospital, Sichuan University, Chengdu, China

**Keywords:** Disability, Long-term care, Predictors, Risk stratification, Geriatrics, Health policy, Public health

## Abstract

This study aimed to develop a deep learning model to predict the risk stratification of all-cause death for older people with disability, providing guidance for long-term care plans. Based on the government-led long-term care insurance program in a pilot city of China from 2017 and followed up to 2021, the study included 42,353 disabled adults aged over 65, with 25,071 assigned to the training set and 17,282 to the validation set. The administrative data (including baseline characteristics, underlying medical conditions, and all-cause mortality) were collected to develop a deep learning model by least absolute shrinkage and selection operator. After a median follow-up time of 14 months, 17,565 (41.5%) deaths were recorded. Thirty predictors were identified and included in the final models for disability-related deaths. Physical disability (mobility, incontinence, feeding), adverse events (pressure ulcers and falls from bed), and cancer were related to poor prognosis. A total of 10,127, 25,140 and 7086 individuals were classified into low-, medium-, and high-risk groups, with actual risk probabilities of death of 9.5%, 45.8%, and 85.5%, respectively. This deep learning model could facilitate the prevention of risk factors and provide guidance for long-term care model planning based on risk stratification.

## Introduction

The rapidly expanding aging population and the improvement of life expectancy have led to an increasing proportion of old people with difficulty living independently^1^. Aging and loss of independence will likely be associated with a high prevalence of multimorbidity and geriatric syndromes (malnutrition, pressure ulcers, cognitive impairment, and falls, etc.). These conditions frequently impact the ability to carry out daily activities, resulting in disability, a significant health issue among old people. Old people with disability have been facing a disproportionately high risk of mortality, hospitalization and care burden, posing substantial challenges to the health care system and support services^2,3^. One demographic study estimated that 108.67–108.79 million persons with disability lived in China in 2020, and this number was projected to increase to 136.24–136.74 million by 2030^4^. Research derived from the China Health and Retirement Longitudinal Study indicates a significant rise in the population with long-term care (LTC) needs from 2011 to 2020^5^. In recent years, the Chinese government has made concerted efforts to establish a contemporary LTC system characterized by high quality, affordability, and alignment with the diverse needs of the aging population. Since 2016, China has initiated the Long-Term Care Insurance (LTCI) program in 15 pilot cities, aiming to offer both financial subsidies and comprehensive care support.

In the mean time, China is also grappling with challenges stemming from a shortage of LTC givers and uneven distribution of LTC resources. Generally, The LTC model can be classified as traditional home care and institutional care. For older people in China, their families have traditionally been the main caregivers. However, partly due to the past one-child policy, rapid urbanization, and increased labor mobility, caregiving by relatives has become increasingly strained as families decrease and dispersed^6^. Although the emotional support provided by relatives may not have decreased across generations, traditional home care could not meet the complex care needs, including rehabilitation therapy, pain control, airway nursing, etc. Meanwhile, the government has promoted the construction of institutional care facilities by preferential policies (such as tax breaks, land allotments, or reduced utility rates), and the number of beds per 1000 people aged 65 or older has more than doubled from 2008 to 2018^6^. Despite this, many beds are not occupied due to high prices, lack of insurance coverage, inadequate services, and inconvenient locations^7,8^. The imbalance between home and institutional care resources calls for innovated planning by governments and health-care providers to ensure adequate, accessible and fair distribution of LTC services.

Therefore, based on the administrative data from an LTCI pilot city from 2017 to 2021, we aim to develop a prognosis model, which could provide LTC model recommendations according to risk stratification and facilitate prevention of risk factors. As far as we know, a prediction model focusing on full-scale functional impairment has not been developed. The traditional Cox proportional hazards model is a semiparametric model that hypothesizes that an individual’s risk exhibits a linear relationship with clinical factors. Given the broad implications and complicated background of disability, prognostic estimation established by a deep learning model may provide more reliable and accurate prediction than simply a linear combination of covariates. Deep learning involves the iteration of artificial intelligence progression that has allowed machines to learn and conclude high-order nonlinear connections between multiple clinical factors and individual consequences in increasingly independent and sophisticated ways^9^. Therefore, this study utilized a deep learning model to predict all-cause mortality and identify risk stratification for older adults with disability.

## Results

### Baseline characteristics and primary outcome

Based on the Chengdu LTCI program initiated in July 2017 and followed up to the date of death or June 2021, participants over 65 years old and identified with disability were included. A total of 42,353 participants were included in the cohort (25,071 for the training set and 17,282 for the validation set), and the baseline characteristics are presented in Supplementary Table S1. At the end of follow-up, 17,565 (41.5%) deaths were observed, with 14,203 (56.7%) in the training set and 3362 (19.5%) in the validation set (Supplementary Tables S2, S3, S4).

The time of disability in survivors and decedents was 48.79 ± 75.66 months vs. 37.40 ± 49.62 months, respectively, and survivors exhibited a younger age (76.08 ± 13.14 years vs. 81.88 ± 9.80 years, p < 0.001); a higher proportion of females [14045 (56.7%) vs. 9558 (54.4%), p < 0.001]; fewer smokers [5250 (21.2%) vs. 3906 (22.2%) p = 0.01]; higher diastolic blood pressure (79.21 ± 45.98 mmHg vs. 75.07 ± 73.26 mmHg, p < 0.001); higher systolic blood pressure (SBP, 138.86 ± 25.42 mmHg vs. 133.43 ± 25.12 mmHg, p < 0.001); fewer adverse events (including scald, falling during walking, falling out of bed, choking on food, and developing pressure scores); more multimorbidities except cancers, fractures, and psychiatric diseases; higher physical ability score (24.68 ± 20.26 vs. 13.71 ± 14.27, p < 0.001); and better cognitive ability score (2.42 ± 1.26 vs. 2.58 ± 1.17, p < 0.001).

### Selected predictors and model establishment

**Figure 1:**
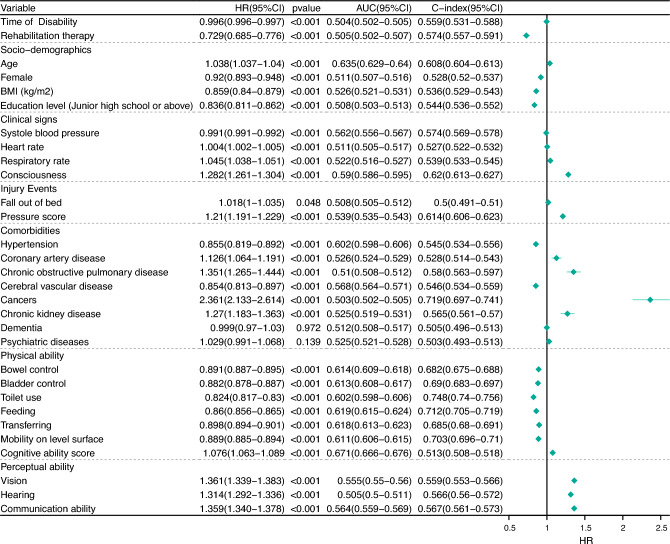
Thirty selected predictors and the HR, AUC, and C-index. *AUC* area under the curve, *C-index* concordance index, *HR* hazard ratio.

Thirty predictors were identified and included in the final cause-specific models for disability-related deaths (Supplementary Fig. S1). The area under the curve (AUC), concordance index (C-index), hazard ratio (HR), and 95% confidence interval (CI) for the predictor variables are presented in Fig. 1.

In detail, older age, higher heart rate, higher respiratory rate, higher level of consciousness, adverse events (falling out of bed and developing pressure sores), and multimorbidities (coronary artery disease, chronic obstructive pulmonary disease (COPD), cancers, psychiatric diseases) were associated with risk of mortality. Rehabilitation therapy, female sex, higher BMI, education level (junior high school or above), SBP, hypertension, cerebral vascular disease, and dementia were associated with positive outcomes. Furthermore, higher scores for physical ability (incontinence, toilet use, feeding, transferring, mobility on level surface) were associated with a better prognosis. The cognitive ability score and perceptual ability exhibited worse prognoses with higher scores.

### Subgroup analysis

**Figure 2:**
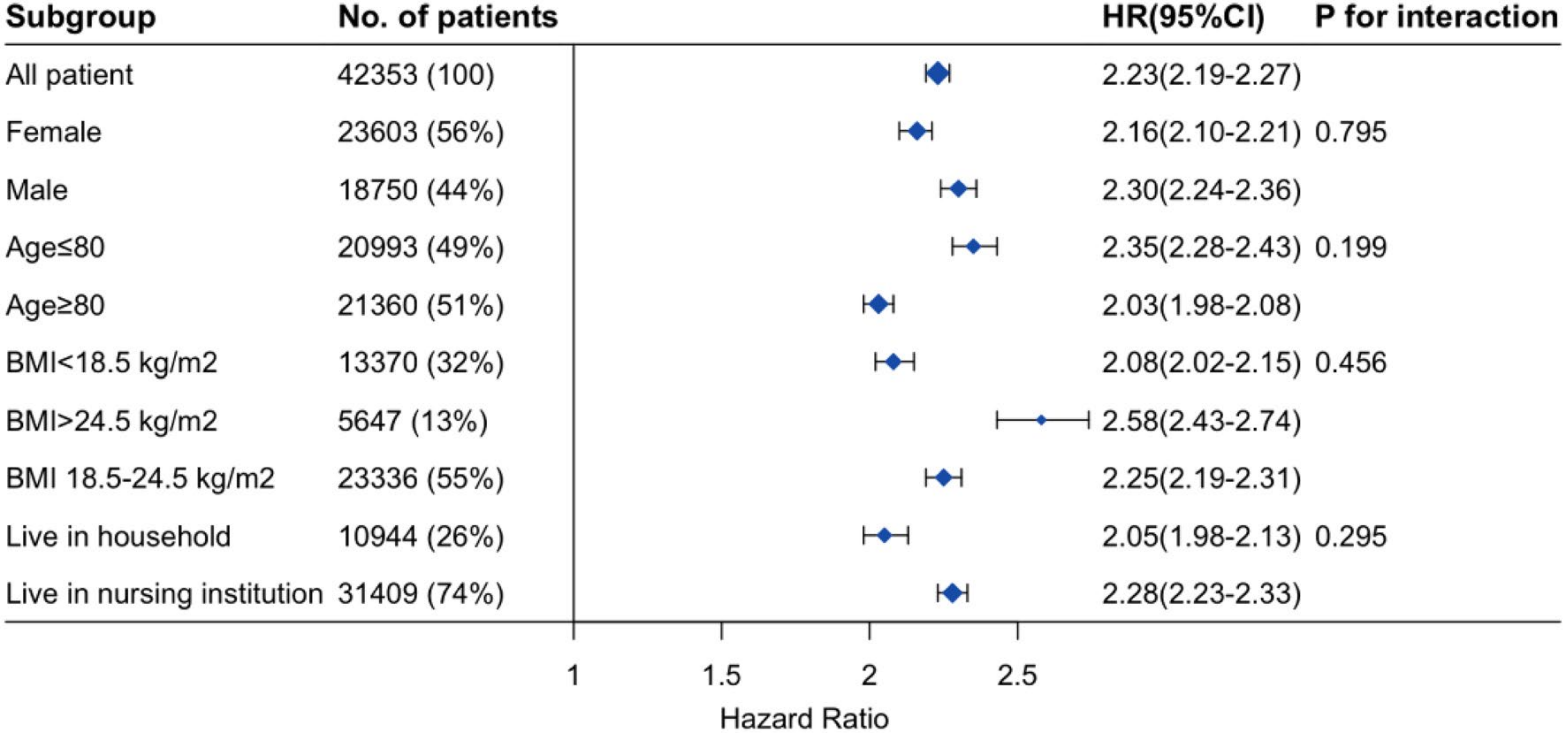
Subgroup analysis and P value for interaction.

In the subgroup analysis, the P value for interaction in subgroups of sex, age, BMI, and care model were 0.795, 0.199, 0.456, and 0.295, respectively. The P value for interaction > 0.05 indicated no interaction between the prediction model and subgroup variables, and the prediction effect was homogeneous across all pre-specified subgroups (Fig. 2).

### Performance of the prediction model

**Figure 3:**
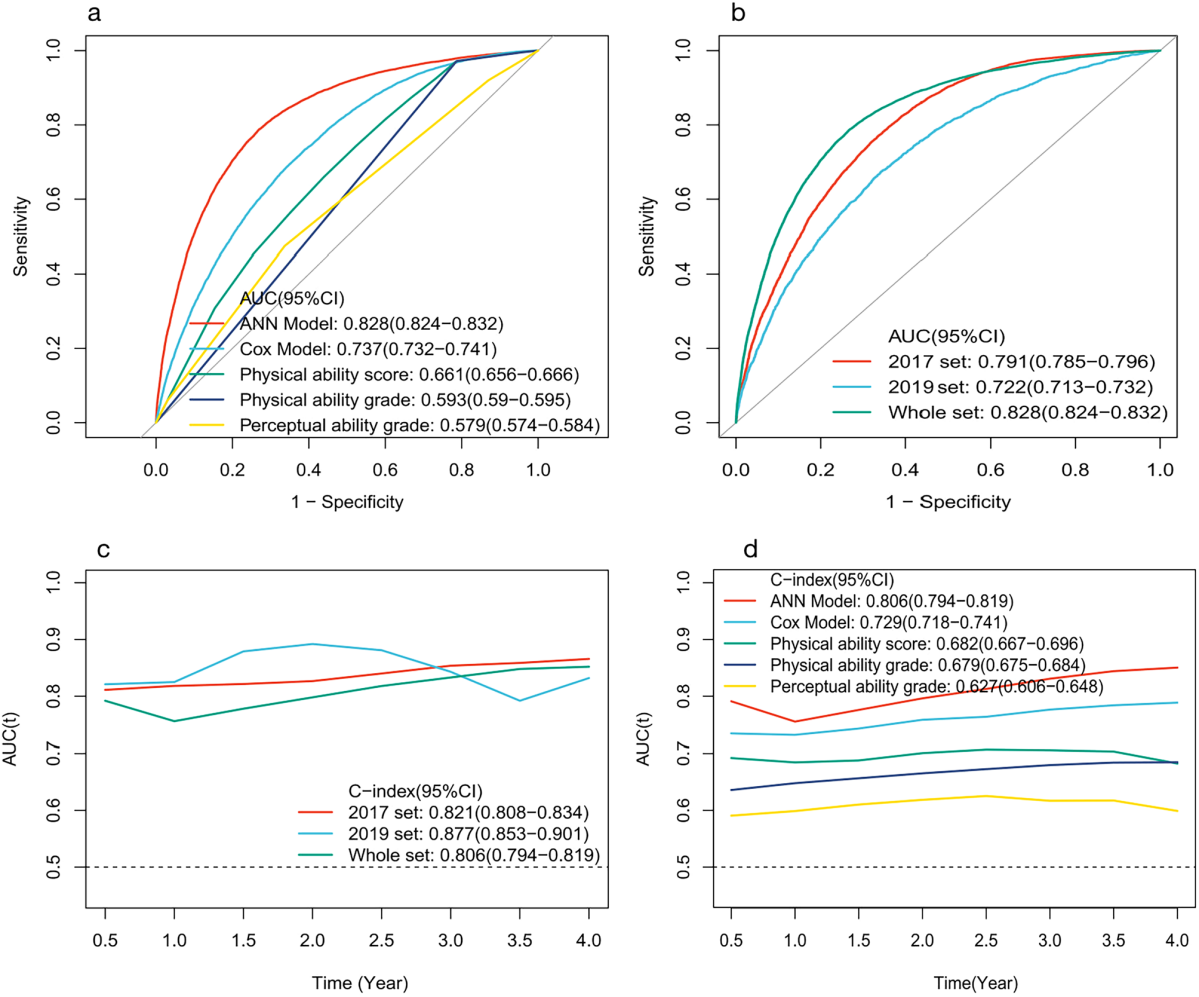
The AUC for the ROC and the time-dependent ROC curve. (**a**) compares the AUCs of the ANN model, classic Cox model, physical ability score, physical ability grade, and perceptual ability grade. (**b**) compares the whole set, validation group (2019 set) and training group (2017 set). (**c**) shows that the AUC of the ANN model was the highest at each point in time. Figure 2d shows the AUC of the whole set, validation group (2019 set) and training group (2017 set) at each point in time. *ANN* artificial neural network, *AUC* area under the curve, *C-index* concordance index.

The C-index and AUC of our deep learning (artificial neural network, ANN) model were 0.806 (95% CI, 0.794–0.819) and 0.828 (95% CI, 0.824–0.832), respectively. Those of the classic Cox model were 0.729 (95% CI, 0.718–0.741) and 0.737 (95% CI, 0.732–0.741), respectively (Fig. 3; Table 1). The comparison between the ANN model and the classic Cox model and the precision-recall curves for the internal validation set are summarized in Table 2. According to the precision-recall curve, the classification performance of the ANN model (AUC = 0.7602) was significantly better than that of the classic Cox model (AUC = 0.6632) (Supplementary Fig. S2). The time-dependent receiver-operator characteristic (ROC) curves are presented in Fig. 3c, revealing that the AUC of the ANN model was the highest at each time point. The AUC and C-index of the ANN model were superior to those of the physical ability score model, physical ability grade model, cognitive ability grade model, and perceptual ability grade model (Fig. 3a,d). The above results showed the predictive performance of the deep learning model was superior than classic Cox model.

**Table 1:** Comparison of the C-index, AUC, and P-AUC between the ANN model and the traditional Cox model.

	C-index		AUC		P-AUC	
	Classic Cox	ANN (P value)	Classic Cox	ANN (P value)	Classic Cox	ANN (P value)
Training set	0.711	0.821 (0.004)	0.790	0.791 (0.009)	0.703	0.704 (0.004)
Validation set	0.757	0.877 (0.002)	0.722	0.722 (0.999)	0.615	0.617 (0.125)
Total	0.729	0.806 (0.029)	0.737	0.828 (0.001)	0.676	0.720 (0.001)

*ANN* artificial neural network, *AUC* area under the curve, *C-index* concordance index, *P-AUC* partial AUC.

**Table 2:** The AUC, C-index, HR &MR recall, and HR recall in training set, validation set and total set.

	Training set	Validation set	Total
No. of patients (deceased)	25,071 (14,203)	17,282 (3362)	42,353 (17,565)
AUC (95% CI)	0.791 (0.785–0.796)	0.722 (0.713–0.732)	0.828 (0.824–0.832)
C-index (95% CI)	0.821 (0.808–0.834)	0.877 (0.853–0.901)	0.806 (0.794–0.819)
HR&MR recall (95% CI)	0.602 (0.598–0.606)	0.567 (0.561–0.574)	0.641 (0.638–0.645)
HR recall (95% CI)	0.587 (0.581–0.593)	0.545 (0.536–0.555)	0.583 (0.578–0.588)

*AUC* area under the curve, *C-index* concordance index, *HR* high risk, *MR* median risk.

### Risk stratification

**Figure 4:**
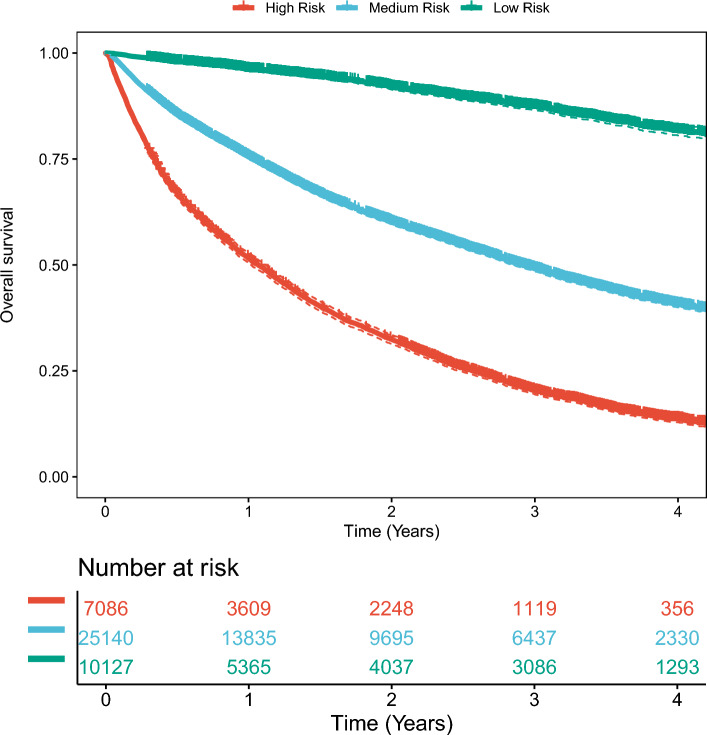
Kaplan–Meier curves of the low-, medium-, and high-risk groups.

A total of 10,127, 25,140 and 7086 patients were classified into low-, medium-, and high-risk groups by deep learning model, with actual risk probabilities of death of 9.5%, 45.8%, and 85.5%, respectively. Kaplan‒Meier curves of these three groups demonstrated statistically significant separation (Fig. 4). The median survival times of the high- and medium-risk groups were 1.07 and 2.92 years, respectively, and the survival time of the low-risk group was more than 4 years. Table 3 lists examples of individuals and their predicted survival time.

**Table 3:** Clinical examples of individuals and their predicted mortality over a 4-year period.

Variables	Patient 1	Patient 2	Patient 3	Patient 4
Age (years)	94	76	70	83
Sex	Female	Female	Female	Male
BMI (kg/m^2^)	< 18.5	> 24.5	> 24.5	18.5–24.5
Endpoint	Survive	Dead	Survive	Dead
Follow-up time (day)	1541	527	1597	596
Time of Disability (month)	15	6	418	69
Rehabilitation therapy	N	Y	N	N
Education level (junior high school or above)	Y	N	Y	N
Systole blood pressure (mmHg)	118	140	108	140
Heart rate (beat per minute)	80	80	86	70
Consciousness	Drowsiness	Lethargy	Conscious	Conscious
Fall out of bed	N	N	N	N
Pressure score	N	N	N	N
Hypertension	Y	Y	N	Y
Chronic obstructive pulmonary disease	N	N	N	N
Cerebral vascular disease	Y	Y	N	N
Cancers	N	N	N	N
Chronic kidney disease	N	N	N	N
Dementia	Y	N	N	Y
Psychiatric diseases	N	N	N	Y
Physical ability				
Bowel control	0	0	5	0
Bladder control	0	0	5	0
Toilet use	0	0	5	5
Feeding	0	0	5	0
Transferring	0	0	5	10
Mobility on level surface	0	0	0	10
Cognitive ability score	2	4	4	3
Perceptual ability				
Vision	Moderate	Moderate	Mild	Robust
Hearing	Mild	Mild	Robust	Mild
Communication ability	Moderate	Severe	Robust	Severe
Survival rate at 1-year (%)	89	55	89	40
Survival rate at 2-year (%)	81	35	81	20
Survival rate at 3-year (%)	75	23	74	10
Survival rate at 4-year (%)	69	15	68	5

*Y* yes, *N* no.

We also conducted risk stratification using traditional Cox regression. A total of 8700, 24,141 and 9512 patients were classified into low-, medium-, and high-risk groups, with actual risk probabilities of death of 9.0%, 43.1%, and 84.2%, respectively. Kaplan–Meier curves of these three groups are illustrated in Supplementary Fig. S3.

## Discussion

This study was the first to use a deep learning model incorporated comprehensive functional assessments, with the purpose of risk stratification in disabled older adults. The key findings of this study were as follows: (1) Physical disability (mobility, incontinence, feeding); coronary artery disease, cancer and COPD; and adverse events (pressure ulcers and falls from bed) were associated with worse outcome among older people, while higher BMI, hypertension, higher SBP, cerebrovascular disease, and rehabilitation therapy were recognized as protective factors. (2) The deep learning survival model demonstrated superior predictive power and helped targeting interventions ranging from the prevention of risk factors to enhancing the quality of care. (3) The deep learning model also offers guidance for LTC service models based on risk stratification.

### Prognostic factors in the deep learning model

Given that some factors are irreversible and unmodifiable, the discussion mainly focused on the reversible factors, including functional decline, geriatric syndromes, and injury events. Unexpectedly, higher BMI, hypertension, and higher SBP tended to be protective against all-cause mortality. This finding was interpreted as the ‘risk factor paradox’, which means reverse associations between the classic cardiovascular risk factors (such as hypertension and obesity) and mortality observed in the oldest old^10,11^. One potential explanation for the inverse association of BMI with mortality is that overweight and obesity may be indicators of better nutritional status^12^. Evidence has supported the survival benefit of higher BMI, with excess adipose tissue serving as an energy reserve and being protective during negative energy balance^11^. However, malnutrition, associated with weight loss and muscle weakness, contributes to the decline in the immune system and makes elderly people more susceptible and vulnerable to infectious disease^13^.

Regarding blood pressure, large population-based longitudinal studies have shown that the association between SBP and the risk of all-cause death exhibits a U-shape^14^. The most likely explanation for increased mortality risk in participants with lower SBP was increased risk for dementia, frailty, and heart failure^15,16^. Some observational cohort studies revealed that increased SBP (> 140 mmHg) was not associated with excess mortality and was even negatively correlated with the risk of death in poor-functioning or severely frail older people^17^. Although debates still exist about the optimal blood pressure level and the associated benefits, more liberal blood pressure management should be adopted in disabled older patients.

Functional loss was generally a risk factor for poor outcomes. Our study found that mobility, incontinence, and feeding were screened in the model and associated with mortality. Studies have shown that confinement to bed is associated with an increased risk of respiratory and infection-related mortality^18^. Urinary or fecal incontinence is associated with a high risk of developing moisture lesions, pressure ulcers and secondary infection. A targeted physical assessment (genital, rectal examination and neurologic evaluation) and avoidance of environmental triggers (infection evaluation, improper medication) are crucial for disabled older patients with incontinence^19^. Feeding difficulty with induced undernutrition in older individuals is clearly related to physical function, health care utilization, and all-cause mortality. Potentially relevant swallowing disorders significantly increase the incidence of aspiration and fatal aspiration pneumonia. Older people with feeding difficulty should undergo further evaluation for undernutrition, possible medical or medication-related issues, dental status, food security, food-related functional status, swallowing ability, and previous dietary restriction^20,21^.

Disabled older people constitute a distinct demographic, distinguished significantly from the general community population. Cerebrovascular disease (stroke, dementia, etc.) serves as an onset causes of disability rather than a risk factor for mortality among this population. As above discussed, malnutrition, infection-related disease, and organ failure were related to all-cause death. The individuals with cerebrovascular disease potentially exhibit better nutritional metabolic statuses with attenuated risk of infections. Secondary prevention drugs for cerebrovascular disease and advanced LTC services have played a pivotal role in decreasing the likelihood of second cardiovascular events attack. These factors collectively contributed to their relatively extended survival time.

In contrast, rehabilitation therapy was associated with a beneficial prognosis. A randomized clinical trial reported that enhanced medical rehabilitation by increasing engagement and intensity of therapy sessions had an estimated 25% greater functional recovery on standard rehabilitation therapy^22^. In addition, an increasing variety of patient assistive technology aids can improve the capacity for activity and/or reduce task demands. The latest released Global Report on Assistive Technology by the WHO shows that almost one-third of people worldwide, including disabled older people, have a need for at least one assistive product, such as wheelchairs, spectacles, and hearing aids^23^. Provision of these products and training personnel to fit, train, adjust and maintain the product are also needed. Specific to disabled older people, interventions targeted at enhancing mobility, feeding, hearing, or vision are essential to improve self-care and decrease adverse events.

An individual’s level of function has been considered a dynamic process determined by health conditions within the context of personal and environmental factors. Most traditional medical prediction models are based on objective indicators, e.g., blood pressure, total cholesterol, certain diseases^24^, specific tumor markers and relevant genes^25^. A small number of studies used function-related indicators, e.g., walking speed, grip strength and subjective symptoms scale, as prognostic indexes in the gerontological field^26,27^. This deep learning model uncovered the nonlinear relation among complicated clinical factors and their hazard ratios. From the results, our deep learning model is significantly superior to the classic Cox model in terms of the C-index and pAUC.

### Guiding long-term care services

The proposed model could assess risk stratification and thus would have important implications for planning LTC. For disabled older adults at high risk of mortality, skilled LTC institutions are more suitable for those who desire symptom control and palliative care. Institutional care facilities provide more professional services (pain control, ventilator support, etc.) along with increasing care intensity. On the one hand, palliative rehabilitation, which is function-directed care for those who have serious and often incurable illnesses marked by intense and dynamic symptoms, psychological stress, and medical morbidity, could vigorously improve the quality of life^28^. Additionally, a relatively shorter period of survival in the institutional care setting contributes to cost savings for both families and governments, preserving financial and care-related resources. For disabled older adults at low- and medium-risk of mortality with relatively longer survival time, community-based medical institutions providing care at home or in community-based settings would be the optimal care settings. Since the health condition of LTC older generation is complex, with a high prevalence of multimorbidity and health disparities between rural and urban areas, hoping to address these challenges by simply expanding the number of workers in institutions is unfeasible. This calls for a move from disease-centred care to person-centred care; care for older people should be primarily community and family based, rather than LTC institution based^29^. Home and community-based services (HCBS) are expected to play an important role in extenuating the financial burden and helping older adults manage their health and maintain their independence. This model is also consistent with older people’s preferences and China’s long tradition of family-based care of older relatives. This dual approach recognizes the diverse needs of older disabled adults and strives to optimize their quality of life while considering the different level of care intensity, care cost and survival time.

In China, initiatives to accelerate the development of the HCBS have been launched across major cities over the past 10 years^8^. HCBS utilization could not only lead to fewer limitations with instrumental activities of daily living, but also delay the progression to a high risk of mortality. As one of the pilots for the LTCI program, our data revealed a considerable proportion of low- and medium-risk populations, for whom HCBS is greatly demanded. This in turn provokes strengthening community-focused care and integrating resources and financial support to provide comprehensive services.

### Strengths and limitations

We have established and validated a deep learning model utilizing standardized functional assessments among disabled older adults for the first time. The risk stratification based on deep learning model simultaneously assists governments in allocating LTC care resources and helps families choose appropriate care models. However, some limitations should be noted. First, the sample was from the LTCI cohort dataset, and we did not perform external validation in populations from different locations in China. Second, since only older people with severe disability were qualified to be granted LTCI, the predictive power of severe disability on mortality may be underestimated to some extent.

## Conclusion

This study developed a deep learning model to predict all-cause mortality in older adults with disabilities. Intervention and research should focus on functional damage (mobility, incontinence, feeding) and the associated harm (infection, malnutrition and adverse events), particularly in the field of rehabilitation and assistive technology. We recommend the group at high-risk choosing professional LTC institutions, and the groups of low- and medium-risk are recommended for HCBS. These recommendations provide a strategic framework for optimizing care delivery and resource allocation in the field of LTC for older adults with disabilities.

## Methods

### Study population and data source

This was a prospective, cohort study based on the Chengdu LTCI program initiated in July 2017 and followed up to the date of death or June 2021.

To reduce economic burdens and improve care quality, China initiated LTCI program in 15 pilot cities since 2016. As one of the pilot cities, Chengdu issued the “Pilot Plan for the Long-Term Care Insurance System” in 2017, which stipulated that people who are unable to take care of themselves and needed LTC due to old age, diseases or disability, could apply for LTCI^30^. The LTCI in Chengdu was funded fully by Chengdu Healthcare Security Administration, and provided monetary reimbursement, basic and social support care services. The basic care services were divided into four categories for beneficiaries, including life care, non-therapeutic care, risk prevention and functional maintenance^31^.

After signing the online informed consent by applicants, evaluation team with 2–3 trained medical workers would obtain the signed informed consent from the applicants or their family members, and then conduct a face-to-face interview and physical examination for applicants (Supplementary Fig. S4)^32^.

Disability status refers to three dimensions (i.e., physical ability, cognitive ability, perceptual ability) and four levels of disability (i.e., robust, mild impairment, moderate impairment, serious impairment). Physical ability was assessed using the activities in daily living (ADL) score, evaluated by the Barthel index. It included ten personal activities, including feeding, bathing, grooming, dressing, bowel and bladder control, toilet use, steps, transfer, and mobility. These activities were assessed on a scale of 0–5 (bathing, grooming), 0–10 (feeding, dressing, bowel control, bladder control, toilet use, stairs), and 0–15 points (transfer, mobility on level surfaces). A higher score indicates a higher level of performing basic ADLs, and the total score ranges from 0–100 points. Cognitive and perception impairment were assessed by the cognitive assessment scale of the LTC program^33^. Cognitive tests focused on memory and the ability to concentrate, while perception tests included vision, hearing, and communication abilities. The details are illustrated in Supplementary Table S5.

The sociodemographics (age, sex, education level, marital status, and caregivers), adverse events in the past month, multimorbidities, rehabilitation therapy, and time of disability, were collected at application. When the applicants applying for LTCI, they must provide the official medical records detailing their medical history and condition. After the preliminary review of the applications, the government will send trained medical workers to their homes or institutions to assess their functional status, as above described. They would measure vital signs and collect drug use during the assessment. The primary endpoint was all-cause mortality. The date of death was extracted from the official death certificates and social insurance system, and validated by Chengdu Healthcare Security Administration. Given the source of administrative data, there was no missing data regarding death. As a supplementary method of follow-up, caregivers were required to submit a monthly 8-s video online to Chengdu Healthcare Security Administration, facilitating face recognition to confirm the participants' survival status. In case a participant failed to submit a video on time, the government would set up an inquiry.

The study was performed in accordance with the Declaration of Helsinki. All participants agreed with the informed consent, which was presented at the beginning of the study. The study was in accordance with STROBE and TRIPOD reporting guidelines. Ethical approval was granted by the institutional ethics review committee of West China Hospital (2021-687).

### Data imputation

Overall, approximately 10% of the data were missing for the covariates, including smoking (9.94%), drinking (9.94%), rehabilitation therapy (9.56%), multiple drug use (8.49%), BMI (8.37%), systolic blood pressure (0.98%), diastolic blood pressure (1.46%), heart rate (0.87%), and respiratory rate (1.75%). Multiple imputation methods were used to handle the missing data^34^. The overall covariates were divided into three groups: numeric covariates, binary covariates and factor covariates. We used the mice function of R package "MICE" to impute the missing data according to different regression methods: predictive mean matching was used to impute numeric features, logistic regression to impute binary variables and Bayesian polytomous regression to impute factor features^35^.

### Selection of predictors

The survivors and decedents were divided into groups by the endpoint of mortality by the end of follow-up. The baseline characteristics were compared between the groups, and the covariates with significant difference were selected as risk factors. Fifty-four baseline clinical features with at least 80% complete data were considered candidate predictors and were used for model establishment. The candidate predictors included sociodemographics, clinical signs, adverse events in the past month, multimorbidities and disability status.

### Deep learning model development

Least absolute shrinkage and selection operator (LASSO) was used to identify predictors with a statistically significance (P < 0.05)^36^. The HRs (95% CI) of selected predictors were estimated by univariate analysis using Cox regression. The total participants were divided into a model training set (2017 cohort) and a validation set (2019 cohort). The training set consisted of participants included since July 2017, and the validation set consisted of those included since July 2019.

We constructed a three-layer feedforward ANN for survival modelling. The network architecture is illustrated in Supplementary Fig. S5. The 30 selected features were fed into the network after data normalization. The network is composed by 4 fully connected layers including 3 hidden layers and 1 output layer. The activation function of the hidden layer was ReLU. The loss function was the negative log partial likelihood under the Cox proportional hazards model. The loss function of the model was defined as following:

$${\text{Loss}}\left( {\uptheta } \right) = - \frac{1}{{{\text{N}}_{{{\text{E}} = 1}} }}\mathop \sum \limits_{{{\text{i}}:{\text{E}}_{{\text{i}}} = 1}} \left( {{\text{h}}^{{\uptheta }} \left( {x_{{\text{i}}} } \right) - \log \mathop \sum \limits_{{{\text{j}} \in {\text{R}}\left( {{\text{T}}_{{\text{i}}} } \right)}} {\text{e}}^{{{\text{h}}^{{\uptheta }} \left( {x_{{\text{i}}} } \right)}} } \right) + {\uplambda } \cdot \left\| {\uptheta } \right\|_{2}^{2}$$

where θ is the parameter of the model to be optimized and ${\text{h}}^{{\uptheta }} \left( {x_{{\text{i}}} } \right)$ is the risk score predicted by the network given input features $x_{{\text{i}}}$. ${\text{N}}_{{{\text{E}} = 1}}$ is the number of decedents. $\log \mathop \sum \nolimits_{{{\text{j}} \in {\text{R}}\left( {{\text{T}}_{{\text{i}}} } \right)}} {\text{e}}^{{{\text{h}}^{{\uptheta }} \left( {x_{{\text{i}}} } \right)}}$ is the logarithmic sum of the risks of all individuals who survived before time $x_{{\text{i}}}$.

To avoid overfitting, we used fivefold nested cross-validation and dropout parameters after each hidden layer. The early stopping strategy = 0.3 was also utilized for regularization purposes. The hyperparameters in our ANN model included the depth and size of the network, dropout rate, learning rate, and weight decay. To tune these hyperparameters, we performed a 1000 iteration random search using the ‘adam’ optimizer and C-index optimization. Output of the network is a single node, which predicts the risk score of all-cause mortality. The network was optimized by gradient descending with gradients estimated by Adam optimizer. Hyperparameters including layer size, learning rate, dropout rate, and training epochs were optimized by Bayesian Hyperparameters Optimization. The optimal parameters are presented in the Supplementary file. The final model was obtained by training the network with the optimal hyperparameters on the training set.

The C-index and AUC for the ROC were evaluated on the model validation cohort to assess predictive performance and ranged from 0.5 (random chance) to 1.0 (perfect prediction)^38^. Time-dependent ROC curves were used to compare the predictive performance at every half-year follow-up.

We further calculated the risk of each individual in the entire training cohort and divided all patients into three groups based on the risk cut-off of 95% sensitivity and 95% specificity of ROC^35^. Individuals with a risk score higher than 95% sensitivity were defined as high-risk group, and those with a risk score lower than 95% specificity were defined as low-risk group. Individuals with risk scores between the two cut-offs were defined as medium-risk group. Finally, a predicted risk value for each LTCI participant was generated based on deep learning model, and a risk estimation was provided.

### Subgroup analysis

Subgroup analysis was performed to identify the stability of the deep learning model and whether the prediction effect is dependent on demographic factors. The following subgroup analyses were conducted: (1) male versus female participants; (2) participants aged < 80 years versus those aged ≥ 80 years; (3) participants with BMI < 18.5 kg/m^2^ versus BMI 18.5–24.5 kg/m^2^ and BMI > 24.5 kg/m^2^; and (4) participants who lived in professional nursing institutions versus those who lived in households. The P value for interaction was determined using Cox regression, and available confounding factors were adjusted.

### Data analysis

Continuous variables were described as mean ± standard deviation and compared using Student’s *t*-test, or described as median (Q25, Q75) and compared using Mann–Whitney tests, according to the distribution. Categorical variables were described as percentages (%) and compared using the chi-square test. The survival time between groups were compared by log rank test. All statistical analyses were conducted with R software (version 4.1.0, Free Software Foundation, Inc., Boston, MA). The R package “survival models” was used to build the ANN model, and the “mlr3” package was used to tune the parameters^37^. The “survcomp” package was used to calculate the consistency C-index, with a larger C-index indicating more accurate predictive power of the model. The ROC curve of the model was calculated and generated using the “pROC” package. The “timeROC” package was used to plot time-dependent ROC curves. Survival curves were drawn by the “survminer” package. Statistical significance was set at 0.05, with two-tailed statistical testing.

### Supplementary Information

Supplementary Information 1.

Supplementary Information 2.

Supplementary Information 3.

## Data Availability

The datasets analyzed during the study are not publicly available due to the confidentiality policy of the Chengdu Insurance system but are available from the corresponding author Qing-Yu Dou upon reasonable request. In addition, this database is open for validation of the results of future studies worldwide through collaboration with the staff of the National Clinical Research Center of Geriatrics in West China Hospital.

## Code availability

The code used in this study for developing the model is provided at https://github.com/Tan-Huaicheng/Deep-learning-for-disability.
