# Supplementary material for: Deep learning model for the prediction of all-cause mortality among long term care people in China: a prospective cohort study
Source: Sci Rep. 2024 Jun 25;14:14639. doi: 10.1038/s41598-024-65601-4 (PMC11199641; doi:10.1038/s41598-024-65601-4)

**Deep learning model for the prediction of all-cause mortality among long term care people in China: a prospective cohort study**

**Author:** Huai-Cheng Tan, MD^1^, Li-Jun Zeng, PhD^2^, Shu-Juan Yang, phD^3,4^, Li-Sha Hou, MD^5^, Jin-Hui Wu, PhD^5^, Xin-Hui Cai, PhD^6^, Fei Heng, PhD^6^, Xu-Yu Gu, PhD^7^, Yue Zhong, MD^8^, Bi-Rong Dong, MD^5^, Qing-Yu Dou, PhD^5 *^

**Appendix The final optimized model parameters** 1

**Supplementary Table S1 Baseline characteristics compared between training and validation sets**  2

**Supplementary Table S2 Baseline characteristics compared between survivors and decedents** 6

**Supplementary Table S3 Baseline characteristics of validation set** 11

**Supplementary Table S4 Baseline characteristics of training set** 16

**Supplementary Table S5 Candidate predictors of mortality** 20

**Supplementary Figure S1 Thirty clinical features identified by LASSO model** 24

**Supplementary Figure S2 Precision-Recall curve of deep learning model and Cox model** 25

**Supplementary Figure S3 Kaplan-Meier curves of the low-, medium-, and high-risk groups, stratified by traditional Cox regression** 26

**Supplementary Figure S4. Flowchart of LTCI application process** 27

**Supplementary Figure S5. Schematic diagram of optimal Artificial Neural Network model** 28

**The final optimized model parameters**

$dropout

[1] 0.1904242

$weight_decay

[1] 0.2379212

$learning_rate

[1] 0.9692816

$num_nodes

[1] 16 38 29

$frac

[1] 0.3

$epochs

[1] 2000

$early_stopping

[1] TRUE

$optimizer

[1] "adam"

$activation

[1] "relu"

**Supplementary Table S1 Baseline characteristics compared between training and validation sets**

|  | Training set (n=25071) | Validation set (n=17282) | P value |
| --- | --- | --- | --- |
| Death (%) | 14203 (56.7) | 3362 (19.5) | <0.001 |
| Follow-up time (month), median (Q25 Q75) | 29 (11, 44) | 8 (6, 12) | <0.001 |
| Time of Disability (month), median (Q25 Q75) | 24 (12,59) | 18(9,40) | <0.001 |
| Rehabilitation therapy (n,%) | 1929 (7.7) | 816 (4.7) | <0.001 |
| Multiple drug use (n,%) | 982 (3.9) | 520 (3.0) | <0.001 |
| Socio-demographics |  |  |  |
| Age (years), , mean (SD) | 79.11 (11.26) | 77.58 (13.41) | <0.001 |
| Female (n, %) | 13887 (55.4) | 9716 (56.2) | 0.093 |
| BMI (n,%) |  |  | <0.001 |
| 0 | 8156 (32.5) | 5214 (30.2) |  |
| 1 | 12807 (51.1) | 10529 (60.9) |  |
| 2 | 4108 (16.4) | 1539 (8.9) |  |
| Education level (Junior high school or above, n,%) | 10197 (40.7) | 5859 (33.9) | <0.001 |
| Marial status (Married, n, %) | 13528 (54.0) | 9639 (55.8) | <0.001 |
| Living in professional nursing institution (n,%) | 18144 (72.4) | 13265 (76.8) | <0.001 |
| Smoking (n,%) | 5636 (22.5) | 3520 (20.4) | <0.001 |
| Drinking (n,%) | 5207 (20.8) | 3231 (18.7) | <0.001 |
| Clinical signs |  |  |  |
| Distole blood pressure (mmHg) | 77.05 (75.09) | 78.12 (17.77) | 0.066 |
| Systole blood pressure (mmHg) | 137.25 (25.37) | 135.68 (25.51) | <0.001 |
| Heart rate (beat per minute) | 80.36 (14.32) | 80.12 (14.20) | 0.09 |
| Respiratory rate | 19.74 (2.22) | 19.74 (2.09) | 0.986 |
| Consciousness (n,%) |  |  | <0.001 |
| 0 | 17784 (70.9) | 15084 (87.3) |  |
| 1 | 2003 (8.0) | 1562 (9.0) |  |
| 2 | 4944 (19.7) | 515 (3.0) |  |
| 3 | 340 (1.4) | 121 (0.7) |  |
| Injury Events |  |  |  |
| Scald (n,%) |  |  | 0.156 |
| 0 | 24480 (97.6) | 16859 (97.6) |  |
| 1 | 354 (1.4) | 263 (1.5) |  |
| 2 | 114 (0.5) | 94 (0.5) |  |
| 3 | 123 (0.5) | 66 (0.4) |  |
| Get lost (n,%) |  |  | <0.001 |
| 0 | 24532 (97.9) | 17035 (98.6) |  |
| 1 | 210 (0.8) | 97 (0.6) |  |
| 2 | 129 ( 0.5) | 53 (0.3) |  |
| 3 | 200 (0.8) | 97 (0.6) |  |
| Fall during walk (n,%) |  |  | <0.001 |
| 0 | 17025 (67.9) | 12293 (71.1) |  |
| 1 | 2663 (10.6) | 1453 (8.4) |  |
| 2 | 1969 (7.9) | 1128 (6.5) |  |
| 3 | 3414 (13.6) | 2408 (13.9) |  |
| Fall out of bed (n,%) |  |  | <0.001 |
| 0 | 20480 (81.7) | 13471 (77.9) |  |
| 1 | 1810 (7.2) | 1407 (8.1) |  |
| 2 | 1197 (4.8) | 922 (5.3) |  |
| 3 | 1584 (6.3) | 1482 (8.6) |  |
| Choke on food (n,%) |  |  | <0.001 |
| 0 | 16474 (65.7) | 12562 (72.7) |  |
| 1 | 1627 (6.5) | 815 (4.7) |  |
| 2 | 1653 (6.6) | 862 (5.0) |  |
| 3 | 5317 (21.2) | 3043 (17.6) |  |
| Suicide attempt (n,%) |  |  | <0.001 |
| 0 | 24571 (98.0) | 17038 (98.6) |  |
| 1 | 240 (1.0) | 107 (0.6) |  |
| 2 | 115 (0.5) | 58 (0.3) |  |
| 3 | 145 (0.6) | 79 (0.5) |  |
| Pressure score (n,%) |  |  | 0.088 |
| 0 | 20264 (80.8) | 14134 (81.8) |  |
| 1 | 2190 (8.7) | 1414 (8.2) |  |
| 2 | 989 (3.9) | 663 (3.8) |  |
| 3 | 1628 (6.5) | 1071 (6.2) |  |
| Multimorbidities |  |  |  |
| Hypertension (n,%) | 1400 (5.6) | 9856 (57.0) | <0.001 |
| Coronary artery disease (n,%) | 714 (2.8) | 3829 (22.2) | <0.001 |
| Heart failure (n,%) | 942 (3.8) | 5147 (29.8) | <0.001 |
| Chronic obstructive pulmonary disease (n,%) | 474 (1.9) | 2276 (13.2) | <0.001 |
| Cerebral vascular disease (n,%) | 1233 (4.9) | 6632 (38.4) | <0.001 |
| Diabetes (n,%) | 708 (2.8) | 3673 (21.3) | <0.001 |
| Cancers (n,%) | 116 (0.5) | 645 (3.7) | <0.001 |
| Chronic kidney disease (n,%) | 521 (2.1) | 1761 (10.2) | <0.001 |
| Dementia (n,%) | 9577 (38.2) | 7905 (45.7) | <0.001 |
| Fracture (n,%) | 299 (1.2) | 94 (0.5) | <0.001 |
| Psychiatric diseases (n,%) | 5117 (20.4) | 1754 (10.1) | <0.001 |
| Physical ability score | 20.39 (18.84) | 19.75 (18.75) | <0.001 |
| Physical ability grade (n,%) |  |  | 0.031 |
| 1 | 680 (2.7) | 429 (2.5) |  |
| 2 | 2694 (10.7) | 1979 (11.5) |  |
| 3 | 21697 (86.5) | 14874 (86.1) |  |
| Bowel control (n,%) |  |  | <0.001 |
| 0 | 11805 (47.1) | 8400 (48.6) |  |
| 5 | 9098 (36.3) | 6702 (38.8) |  |
| 10 | 4168 (16.6) | 2180 (12.6) |  |
| Bladder control (n,%) |  |  | <0.001 |
| 0 | 13699 (54.6) | 9667 (55.9) |  |
| 5 | 8217 (32.8) | 6020 (34.8) |  |
| 10 | 3155 (12.6) | 1595 ( 9.2) |  |
| Grooming =5 (n,%) | 2558 (10.2) | 3827 (22.1) | <0.001 |
| Toilet use (n,%) |  |  | <0.001 |
| 0 | 17793 (71.0) | 12438 (72.0) |  |
| 5 | 7020 (28.0) | 4610 (26.7) |  |
| 10 | 258 ( 1.0) | 234 ( 1.4) |  |
| Feeding (n,%) |  |  | 0.088 |
| 0 | 13702 (54.7) | 9357 (54.1) |  |
| 5 | 9927 (39.6) | 6994 (40.5) |  |
| 10 | 1442 ( 5.8) | 931 (5.4) |  |
| Transferring (n,%) |  |  | <0.001 |
| 0 | 8667 (34.6) | 5974 (34.6) |  |
| 5 | 9435 (37.6) | 7905 (45.7) |  |
| 10 | 6354 (25.3) | 3188 (18.4) |  |
| 15 | 615 ( 2.5) | 215 (1.2) |  |
| Mobility on level surface (n,%) |  |  | <0.001 |
| 0 | 15097 (60.2) | 10599 (61.3) |  |
| 5 | 5891 (23.5) | 4032 (23.3) |  |
| 10 | 3613 (14.4) | 2425 (14.0) |  |
| 15 | 470 ( 1.9) | 226 (1.3) |  |
| Dressing (n,%) |  |  | 0.01 |
| 0 | 19174 (76.5) | 13012 (75.3) |  |
| 5 | 5466 (21.8) | 3933 (22.8) |  |
| 10 | 431 (1.7) | 337 (2.0) |  |
| Stairs (n,%) |  |  | <0.001 |
| 0 | 22840 (91.1) | 16304 (94.3) |  |
| 5 | 2138 (8.5) | 942 (5.5) |  |
| 10 | 93 (0.4) | 36 (0.2) |  |
| Bathing=5 (n,%) | 142 (0.6) | 102 (0.6) | 0.8 |
| Cognitive ability score, mean (SD) | 2.45 (1.17) | 2.54 (1.30) | <0.001 |
| Cognitive ability grade (n,%) |  |  | <0.001 |
| 0 | 515 (2.1) | 1359 (7.9) |  |
| 1 | 4530 (18.1) | 8353 (48.3) |  |
| 2 | 15532 (62.0) | 3690 (21.4) |  |
| 3 | 4494 (17.9) | 3880 (22.5) |  |
| Perceptual ability grade (n,%) |  |  | <0.001 |
| 0 | 3975 (15.9) | 658 (3.8) |  |
| 1 | 11886 (47.4) | 9164 (53.0) |  |
| 2 | 8159 (32.5) | 6759 (39.1) |  |
| 3 | 1051 ( 4.2) | 701 (4.1) |  |
| Perceptual ability |  |  |  |
| Vision (n,%) |  |  | <0.001 |
| 0 | 2379 ( 9.5) | 1351 ( 7.8) |  |
| 1 | 10875 (43.4) | 7057 (40.8) |  |
| 2 | 7545 (30.1) | 6205 (35.9) |  |
| 3 | 4272 (17.0) | 2669 (15.4) |  |
| Hearing (n,%) |  |  | <0.001 |
| 0 | 7859 (31.3) | 3849 (22.3) |  |
| 1 | 7810 (31.2) | 6121 (35.4) |  |
| 2 | 7845 (31.3) | 6207 (35.9) |  |
| 3 | 1557 ( 6.2) | 1105 ( 6.4) |  |
| Communication ability (n,%) |  |  | <0.001 |
| 0 | 4051 (16.2) | 3801 (22.0) |  |
| 1 | 6985 (27.9) | 7620 (44.1) |  |
| 2 | 6682 (26.7) | 1611 ( 9.3) |  |
| 3 | 7353 (29.3) | 4250 (24.6) |  |

**Supplementary Table S2 Baseline characteristics compared between survivors and decedents**

| **Variables** | **Survivors (n=24788)** | **Decedents (n=17565)** | **P value** |
| --- | --- | --- | --- |
| Follow-up time (month),  median (Q25, Q75) | 22 (8, 45) | 11 (4, 23) | <0.001 |
| Time of Disability (month),  median (Q25, Q75) | 24 (10, 55) | 24 (10, 48) | <0.001 |
| Rehabilitation therapy (n,%) | 1702 (6.9) | 1043 (5.9) | <0.001 |
| Multiple drug use (n,%) | 879 ( 3.5) | 623 ( 3.5) | 1.000 |
| Socio-demographics |  |  |  |
| Age (years) | 76.08 ±13.14 | 81.88 ±9.80 | <0.001 |
| Female (n, %) | 14045 (56.7) | 9558 (54.4) | <0.001 |
| BMI (n,%) |  |  | <0.001 |
| 0 | 7326 (29.6) | 6044 (34.4) |  |
| 1 | 14007 (56.5) | 9329 (53.1) |  |
| 2 | 3455 (13.9) | 2192 (12.5) |  |
| Education level (Junior high school or above, n,%) | 9560 (38.6) | 6496 (37.0) | 0.001 |
| Marial status (Married, n, %) | 14391 (58.1) | 8776 (50.0) | <0.001 |
| Living in professional nursing institution (n,%) | 19415 (78.3) | 11994 (68.3) | <0.001 |
| Smoking (n,%) | 5250 (21.2) | 3906 (22.2) | 0.010 |
| Drinking (n,%) | 4861 (19.6) | 3577 (20.4) | 0.057 |
| Clinical signs |  |  |  |
| Distole blood pressure (mmHg) | 79.21 ±45.98 | 75.07 ±73.26 | <0.001 |
| Systole blood pressure (mmHg) | 138.86 ±25.42 | 133.43 ±25.12 | <0.001 |
| Heart rate (beat per minute) | 80.00 ±13.85 | 80.63 ±14.83 | <0.001 |
| Respiratory rate | 19.66 ±2.06 | 19.87 ±2.31 | <0.001 |
| Consciousness (n,%) |  |  | <0.001 |
| 0 | 21045 (84.9) | 11823 (67.3) |  |
| 1 | 1592 (6.4) | 1973 (11.2) |  |
| 2 | 2008 (8.1) | 3451 (19.6) |  |
| 3 | 143 (0.6) | 318 (1.8) |  |
| Injury Events |  |  |  |
| Scald (n,%) |  |  | <0.001 |
| 0 | 24122 (97.3) | 17217 (98.0) |  |
| 1 | 403 (1.6) | 214 (1.2) |  |
| 2 | 138 (0.6) | 70 (0.4) |  |
| 3 | 125 (0.5) | 64 (0.4) |  |
| Get lost (n,%) |  |  | 0.812 |
| 0 | 24340 (98.2) | 172287(98.1) |  |
| 1 | 174 ( 0.7) | 133 (0.8) |  |
| 2 | 102 ( 0.4) | 80 (0.5) |  |
| 3 | 172 ( 0.7) | 125 (0.7) |  |
| Fall during walk (n,%) |  |  | <0.001 |
| 0 | 16808 (67.8) | 12510 (71.2) |  |
| 1 | 2564 (10.3) | 1552 (8.8) |  |
| 2 | 1942 (7.8) | 1155 (6.6) |  |
| 3 | 3474 (14.0) | 2348 (13.4) |  |
| Fall out of bed (n,%) |  |  | <0.001 |
| 0 | 19675 (79.4) | 14276 (81.3) |  |
| 1 | 2013 (8.1) | 1204 (6.9) |  |
| 2 | 1293 (5.2) | 826 (4.7) |  |
| 3 | 1807 (7.3) | 1259 (7.2) |  |
| Choke on food (n,%) |  |  | <0.001 |
| 0 | 17442 (70.4) | 11594 (66.0) |  |
| 1 | 1384 (5.6) | 1058 (6.0) |  |
| 2 | 1409 (5.7) | 1108 (6.3) |  |
| 3 | 4554 (18.4) | 3806 (21.7) |  |
| Suicide attempt (n,%) |  |  | 0.993 |
| 0 | 24354 (98.2) | 17255 (98.2) |  |
| 1 | 204 ( 0.8) | 143 ( 0.8) |  |
| 2 | 101 ( 0.4) | 72 ( 0.4) |  |
| 3 | 129 ( 0.5) | 95 ( 0.5) |  |
| Pressure score (n,%) |  |  | <0.001 |
| 0 | 20907 (84.3) | 13491 (76.8) |  |
| 1 | 1808 (7.3) | 1796 (10.2) |  |
| 2 | 832 ( 3.4) | 820 (4.7) |  |
| 3 | 1241 (5.0) | 1458 (8.3) |  |
| Multimorbidities |  |  |  |
| Hypertension (n,%) | 8674 (35.0) | 2582 (14.7) | <0.001 |
| Coronary artery disease (n,%) | 3201 (12.9) | 1342 (7.6) | <0.001 |
| Heart failure (n,%) | 4318 (17.4) | 1771 (10.1) | <0.001 |
| Chronic obstructive pulmonary disease (n,%) | 1816 (7.3) | 934 ( 5.3) | <0.001 |
| Cerebral vascular disease (n,%) | 6006 (24.2) | 1859 (10.6) | <0.001 |
| Diabetes (n,%) | 3175 (12.8) | 1206 (6.9) | <0.001 |
| Cancers (n,%) | 380 (1.5) | 381 (2.2) | <0.001 |
| Chronic kidney disease (n,%) | 1479 ( 6.0) | 803 ( 4.6) | <0.001 |
| Dementia (n,%) | 10487 (42.3) | 6995 (39.8) | <0.001 |
| Fracture (n,%) | 201 (0.8) | 192 (1.1) | 0.003 |
| Psychiatric diseases (n,%) | 3514 (14.2) | 3357 (19.1) | <0.001 |
| Physical ability score | 24.68 ±20.26 | 13.71 ±14.27 | <0.001 |
| Physical ability grade (n,%) |  |  | <0.001 |
| 1 | 1031 ( 4.2) | 78 ( 0.4) |  |
| 2 | 4256 (17.2) | 417 ( 2.4) |  |
| 3 | 19501 (78.7) | 17070 (97.2) |  |
| Physical ability |  |  |  |
| Bowel control (n,%) |  |  | <0.001 |
| 0 | 9788 (39.5) | 10417 (59.3) |  |
| 5 | 10191 (41.1) | 5609 (31.9) |  |
| 10 | 4809 (19.4) | 1539 (8.8) |  |
| Bladder control (n,%) |  |  | <0.001 |
| 0 | 11555 (46.6) | 11811 (67.2) |  |
| 5 | 9552 (38.5) | 4685 (26.7) |  |
| 10 | 3681 (14.8) | 1069 (6.1) |  |
| Grooming (n,%) |  |  | <0.001 |
| 5 | 5171 (20.9) | 1214 (6.9) |  |
| Toilet use (n,%) |  |  | <0.001 |
| 0 | 15652 (63.1) | 14579 (83.0) |  |
| 5 | 8676 (35.0) | 2954 (16.8) |  |
| 10 | 460 (1.9) | 32 (0.2) |  |
| Feeding (n,%) |  |  | <0.001 |
| 0 | 11196 (45.2) | 11863 (67.5) |  |
| 5 | 11662 (47.0) | 5259 (29.9) |  |
| 10 | 1930 (7.8) | 443 (2.5) |  |
| Transferring (n,%) |  |  | <0.001 |
| 0 | 6711 (27.1) | 7930 (45.1) |  |
| 5 | 10550 (42.6) | 6790 (38.7) |  |
| 10 | 6799 (27.4) | 2743 (15.6) |  |
| 15 | 728 (2.9) | 102 (0.6) |  |
| Mobility on level surface (n,%) |  |  | <0.001 |
| 0 | 13054 (52.7) | 12642 (72.0) |  |
| 5 | 6362 (25.7) | 3561 (20.3) |  |
| 10 | 4775 (19.3) | 1263 (7.2) |  |
| 15 | 597 ( 2.4) | 99 (0.6) |  |
| Dressing (n,%) |  |  | <0.001 |
| 0 | 17166 (69.3) | 15020 (85.5) |  |
| 5 | 6938 (28.0) | 2461 (14.0) |  |
| 10 | 684 (2.8) | 84 (0.5) |  |
| Stairs (n,%) |  |  | <0.001 |
| 0 | 22206 (89.6) | 16938 (96.4) |  |
| 5 | 2475 (10.0) | 605 (3.4) |  |
| 10 | 107 (0.4) | 22 (0.1) |  |
| Bathing (n,%) |  |  | <0.001 |
| 5 | 224 (0.9) | 20 (0.1) |  |
| Cognitive ability score | 2.42 ±1.26 | 2.58 ±1.17 | <0.001 |
| Cognitive ability grade (n,%) |  |  | <0.001 |
| 0 | 1581 (6.4) | 293 ( 1.7) |  |
| 1 | 9350 (37.7) | 3533 (20.1) |  |
| 2 | 9123 (36.8) | 10099 (57.5) |  |
| 3 | 4734 (19.1) | 3640 (20.7) |  |
| Perceptual ability grade (n,%) |  |  | <0.001 |
| 0 | 3227 (13.0) | 1406 (8.0) |  |
| 1 | 13216 (53.3) | 7834 (44.6) |  |
| 2 | 7624 (30.8) | 7294 (41.5) |  |
| 3 | 721 (2.9) | 1031 (5.9) |  |
| Perceptual ability |  |  |  |
| Vision (n,%) |  |  | <0.001 |
| 0 | 2640 (10.7) | 1090 (6.2) |  |
| 1 | 11297 (45.6) | 6635 (37.8) |  |
| 2 | 7611 (30.7) | 6139 (35.0) |  |
| 3 | 3240 (13.1) | 3701 (21.1) |  |
| Hearing (n,%) |  |  | <0.001 |
| 0 | 7912 (31.9) | 3796 (21.6) |  |
| 1 | 8458 (34.1) | 5473 (31.2) |  |
| 2 | 7195 (29.0) | 6857 (39.0) |  |
| 3 | 1223 ( 4.9) | 1439 ( 8.2) |  |
| Communication ability (n,%) |  |  | <0.001 |
| 0 | 6060 (24.4) | 1792 (10.2) |  |
| 1 | 9647 (38.9) | 4958 (28.2) |  |
| 2 | 3826 (15.4) | 4467 (25.4) |  |
| 3 | 5255 (21.2) | 6348 (36.1) |  |

**Supplementary Table S3 Baseline characteristics of validation set**

| **Variables** | **Survivors (n=13920)** | **Decedents (n=3362)** | **P value** |
| --- | --- | --- | --- |
| Follow-up time (year),  median (Q25, Q75) | 9 (7, 13) | 5 (2, 9) | <0.001 |
| Time of Disability (month),  median (Q25, Q75) | 21 (9, 48) | 12 (8, 27) | <0.001 |
| Rehabilitation therapy (n,%) | 664 ( 4.7) | 155 ( 4.6) | 0.769 |
| Multiple drug use (n,%) | 424 ( 3.0) | 96 ( 2.9) | 0.600 |
| Socio-demographics |  |  |  |
| Age (years) | 76.58 ±13.81 | 81.74 ±10.61 | <0.001 |
| Female (n, %) | 7891 (56.7) | 1825 (54.3) | 0.012 |
| BMI (n,%) |  |  | 0.004 |
| 0 | 4121 (29.6) | 1093 (32.5) |  |
| 1 | 8544 (61.4) | 1985 (59.0) |  |
| 2 | 1255 ( 9.0) | 284 ( 8.4) |  |
| Education level (Junior high school or above, n,%) | 4750 (34.1) | 1109 (33.0) | 0.219 |
| Marial status (Married, n, %) | 7961 (57.2) | 1678 (49.9) | <0.001 |
| Living in professional nursing institution (n,%) | 10874 (78.1) | 2391 (71.1) | <0.001 |
| Smoking (n,%) | 2836 (20.4) | 684 (20.3) | 0.990 |
| Drinking (n,%) | 2611 (18.8) | 620 (18.4) | 0.691 |
| Clinical signs |  |  |  |
| Diastole blood pressure (mmHg) | 79.00 ±16.75 | 74.49 ±21.08 | <0.001 |
| Systole blood pressure (mmHg) | 136.96 ±25.41 | 130.39 ±25.22 | <0.001 |
| Heart rate (beat per minute) | 79.78 ±13.85 | 81.53 ±15.47 | <0.001 |
| Respiratory rate | 19.68 ±2.05 | 20.00 ±2.23 | <0.001 |
| Consciousness (n,%) |  |  | <0.001 |
| 0 | 12422 (89.2) | 2662 (79.2) |  |
| 1 | 1081 ( 7.8) | 481 (14.3) |  |
| 2 | 343 ( 2.5) | 172 ( 5.1) |  |
| 3 | 74 ( 0.5) | 47 ( 1.4) |  |
| Injury Events |  |  |  |
| Scald (n,%) |  |  | 0.826 |
| 0 | 13575 (97.5) | 3284 (97.7) |  |
| 1 | 212 ( 1.5) | 51 ( 1.5) |  |
| 2 | 77 ( 0.6) | 17 ( 0.5) |  |
| 3 | 56 ( 0.4) | 10 ( 0.3) |  |
| Get lost (n,%) |  |  | 0.759 |
| 0 | 13719 (98.6) | 3316 (98.6) |  |
| 1 | 76 ( 0.5) | 21 ( 0.6) |  |
| 2 | 45 ( 0.3) | 8 ( 0.2) |  |
| 3 | 80 ( 0.6) | 17 ( 0.5) |  |
| Fall during walk (n,%) |  |  | 0.009 |
| 0 | 9837 (70.7) | 2456 (73.1) |  |
| 1 | 1196 ( 8.6) | 257 ( 7.6) |  |
| 2 | 942 ( 6.8) | 186 ( 5.5) |  |
| 3 | 1945 (14.0) | 463 (13.8) |  |
| Fall out of bed (n,%) |  |  | 0.789 |
| 0 | 10866 (78.1) | 2605 (77.5) |  |
| 1 | 1133 ( 8.1) | 274 ( 8.1) |  |
| 2 | 742 ( 5.3) | 180 ( 5.4) |  |
| 3 | 1179 ( 8.5) | 303 ( 9.0) |  |
| Choke on food (n,%) |  |  | 0.108 |
| 0 | 10155 (73.0) | 2407 (71.6) |  |
| 1 | 664 ( 4.8) | 151 ( 4.5) |  |
| 2 | 698 ( 5.0) | 164 ( 4.9) |  |
| 3 | 2403 (17.3) | 640 (19.0) |  |
| Suicide attempt (n,%) |  |  | 0.624 |
| 0 | 13727 (98.6) | 3311 (98.5) |  |
| 1 | 85 ( 0.6) | 22 ( 0.7) |  |
| 2 | 43 ( 0.3) | 15 ( 0.4) |  |
| 3 | 65 ( 0.5) | 14 ( 0.4) |  |
| Pressure score (n,%) |  |  | <0.001 |
| 0 | 11601 (83.3) | 2533 (75.3) |  |
| 1 | 1050 ( 7.5) | 364 (10.8) |  |
| 2 | 493 ( 3.5) | 170 ( 5.1) |  |
| 3 | 776 ( 5.6) | 295 ( 8.8) |  |
| Multimorbidities |  |  |  |
| Hypertension (n,%) | 8062 (57.9) | 1794 (53.4) | <0.001 |
| Coronary artery disease (n,%) | 2944 (21.1) | 885 (26.3) | <0.001 |
| Heart failure (n,%) | 3962 (28.5) | 1185 (35.2) | <0.001 |
| Chronic obstructive pulmonary disease (n,%) | 1649 (11.8) | 627 (18.6) | <0.001 |
| Cerebral vascular disease (n,%) | 5483 (39.4) | 1149 (34.2) | <0.001 |
| Diabetes (n,%) | 2895 (20.8) | 778 (23.1) | 0.003 |
| Cancers (n,%) | 349 ( 2.5) | 296 ( 8.8) | <0.001 |
| Chronic kidney disease (n,%) | 1300 ( 9.3) | 461 (13.7) | <0.001 |
| Dementia (n,%) | 6393 (45.9) | 1512 (45.0) | 0.329 |
| Fracture (n,%) | 70 ( 0.5) | 24 ( 0.7) | 0.173 |
| Psychiatric diseases (n,%) | 1426 (10.2) | 328 ( 9.8) | 0.418 |
| Physical ability score | 21.48 ±19.20 | 12.56 ±14.77 | <0.001 |
| Physical ability grade (n,%) |  |  | <0.001 |
| 1 | 403 ( 2.9) | 26 ( 0.8) |  |
| 2 | 1851 (13.3) | 128 ( 3.8) |  |
| 3 | 11666 (83.2) | 3208 (95.4) |  |
| Physical ability |  |  |  |
| Bowel control (n,%) |  |  | <0.001 |
| 0 | 6260 (45.0) | 2140 (63.7) |  |
| 5 | 5684 (40.8) | 1018 (30.3) |  |
| 10 | 1976 (14.2) | 204 ( 6.1) |  |
| Bladder control (n,%) |  |  | <0.001 |
| 0 | 7293 (52.4) | 2374 (70.6) |  |
| 5 | 5173 (37.2) | 847 (25.2) |  |
| 10 | 1454 (10.4) | 141 ( 4.2) |  |
| Grooming (n,%) |  |  | <0.001 |
| 5 | 3430 (24.6) | 397 (11.8) |  |
| Toilet use (n,%) |  |  | <0.001 |
| 0 | 9584 (68.9) | 2854 (84.9) |  |
| 5 | 4119 (29.6) | 491 (14.6) |  |
| 10 | 217 ( 1.6) | 17 ( 0.5) |  |
| Feeding (n,%) |  |  | <0.001 |
| 0 | 7007 (50.3) | 2350 (69.9) |  |
| 5 | 6061 (43.5) | 933 (27.8) |  |
| 10 | 852 ( 6.1) | 79 ( 2.3) |  |
| Transferring (n,%) |  |  | <0.001 |
| 0 | 4332 (31.1) | 1642 (48.8) |  |
| 5 | 6549 (47.0) | 1356 (40.3) |  |
| 10 | 2842 (20.4) | 346 (10.3) |  |
| 15 | 197 ( 1.4) | 18 ( 0.5) |  |
| Mobility on level surface (n,%) |  |  | <0.001 |
| 0 | 8119 (58.3) | 2480 (73.8) |  |
| 5 | 3377 (24.3) | 655 (19.5) |  |
| 10 | 2218 (15.9) | 207 ( 6.2) |  |
| 15 | 206 ( 1.5) | 20 ( 0.6) |  |
| Dressing (n,%) |  |  | <0.001 |
| 0 | 10166 (73.0) | 2846 (84.7) |  |
| 5 | 3440 (24.7) | 493 (14.7) |  |
| 10 | 314 ( 2.3) | 23 ( 0.7) |  |
| Stairs (n,%) |  |  | <0.001 |
| 0 | 13041 (93.7) | 3263 (97.1) |  |
| 5 | 848 ( 6.1) | 94 ( 2.8) |  |
| 10 | 31 ( 0.2) | 5 ( 0.1) |  |
| Bathing (n,%) |  |  | <0.001 |
| 5 | 99 ( 0.7) | 3 ( 0.1) |  |
| Cognitive ability score | 2.47 ±1.29 | 2.82 ±1.29 | <0.001 |
| Cognitive ability grade (n,%) |  |  | <0.001 |
| 0 | 1208 ( 8.7) | 137 ( 4.1) |  |
| 1 | 6960 (50.0) | 1393 (41.4) |  |
| 2 | 2922 (21.0) | 768 (22.8) |  |
| 3 | 2830 (20.3) | 1050 (31.2) |  |
| Perceptual ability grade (n,%) |  |  | <0.001 |
| 0 | 599 ( 4.3) | 59 ( 1.8) |  |
| 1 | 7728 (55.5) | 1436 (42.7) |  |
| 2 | 5113 (36.7) | 1646 (49.0) |  |
| 3 | 480 ( 3.4) | 221 ( 6.6) |  |
| Perceptual ability |  |  |  |
| Vision (n,%) |  |  | <0.001 |
| 0 | 1191 ( 8.6) | 160 ( 4.8) |  |
| 1 | 5942 (42.7) | 1115 (33.2) |  |
| 2 | 4822 (34.6) | 1383 (41.1) |  |
| 3 | 1965 (14.1) | 704 (20.9) |  |
| Hearing (n,%) |  |  | <0.001 |
| 0 | 3365 (24.2) | 484 (14.4) |  |
| 1 | 5048 (36.3) | 1073 (31.9) |  |
| 2 | 4709 (33.8) | 1498 (44.6) |  |
| 3 | 798 ( 5.7) | 307 ( 9.1) |  |
| Communication ability (n,%) |  |  | <0.001 |
| 0 | 3395 (24.4) | 406 (12.1) |  |
| 1 | 6184 (44.4) | 1436 (42.7) |  |
| 2 | 1219 ( 8.8) | 392 (11.7) |  |
| 3 | 3122 (22.4) | 1128 (33.6) |  |

**Supplementary Table S4 Baseline characteristics of training set**

| **Variables** | **Survivors (n=10868)** | **Decedents (n=14203)** | **P value** |
| --- | --- | --- | --- |
| Follow-up time (month),  median (Q25, Q75) | 45 (37, 49) | 13 (5, 25) | <0.001 |
| Time of Disability (month),  median (Q25, Q75) | 29 (12, 60) | 24 (12, 48) | <0.001 |
| Rehabilitation therapy (n,%) | 1041 ( 9.6) | 888 ( 6.3) | <0.001 |
| Multiple drug use (n,%) | 455 ( 4.2) | 527 ( 3.7) | 0.058 |
| Socio-demographics |  |  |  |
| Age (years) | 75.44 ±12.19 | 81.92 ±9.59 | <0.001 |
| Female (n, %) | 6154 (56.6) | 7733 (54.4) | 0.001 |
| BMI (n,%) |  |  | <0.001 |
| 0 | 3205 (29.5) | 4951 (34.9) |  |
| 1 | 5463 (50.3) | 7344 (51.7) |  |
| 2 | 2200 (20.2) | 1908 (13.4) |  |
| Education level (Junior high school or above, n,%) | 4810 (44.3) | 5387 (37.9) | <0.001 |
| Marial status (Married, n, %) | 6430 (59.2) | 7098 (50.0) | <0.001 |
| Living in professional nursing institution (n,%) | 8541 (78.6) | 9603 (67.6) | <0.001 |
| Smoking (n,%) | 2414 (22.2) | 3222 (22.7) | 0.382 |
| Drinking (n,%) | 2250 (20.7) | 2957 (20.8) | 0.834 |
| Clinical signs |  |  |  |
| Diastole blood pressure (mmHg) | 79.47 ±66.80 | 75.20 ±80.82 | <0.001 |
| Systole blood pressure (mmHg) | 141.30 ±25.22 | 134.16 ±25.05 | <0.001 |
| Heart rate (beat per minute) | 80.28 ±13.84 | 80.42 ±14.67 | 0.434 |
| Respiratory rate | 19.62 ±2.07 | 19.84 ±2.33 | <0.001 |
| Consciousness (n,%) |  |  | <0.001 |
| 0 | 8642 (79.4) | 9163 (64.5) |  |
| 1 | 511 ( 4.7) | 1492 (10.5) |  |
| 2 | 1665 (15.3) | 3279 (23.1) |  |
| 3 | 69 ( 0.6) | 271 ( 1.9) |  |
| Injury Events |  |  |  |
| Scald (n,%) |  |  | <0.001 |
| 0 | 10547 (97.0) | 13933 (98.1) |  |
| 1 | 191 ( 1.8) | 163 ( 1.1) |  |
| 2 | 61 ( 0.6) | 53 ( 0.4) |  |
| 3 | 69 ( 0.6) | 54 ( 0.4) |  |
| Get lost (n,%) |  |  | 0.664 |
| 0 | 10621 (97.7) | 13911 (97.9) |  |
| 1 | 98 ( 0.9) | 112 ( 0.8) |  |
| 2 | 57 ( 0.5) | 72 ( 0.5) |  |
| 3 | 92 ( 0.8) | 108 ( 0.8) |  |
| Fall during walk (n,%) |  |  | <0.001 |
| 0 | 6971 (64.1) | 10054 (70.8) |  |
| 1 | 1368 (12.6) | 1295 ( 9.1) |  |
| 2 | 1000 ( 9.2) | 969 ( 6.8) |  |
| 3 | 1529 (14.1) | 1885 (13.3) |  |
| Fall out of bed (n,%) |  |  | <0.001 |
| 0 | 8809 (81.1) | 11671 (82.2) |  |
| 1 | 880 ( 8.1) | 930 ( 6.5) |  |
| 2 | 551 ( 5.1) | 646 ( 4.5) |  |
| 3 | 628 ( 5.8) | 956 ( 6.7) |  |
| Choke on food (n,%) |  |  | <0.001 |
| 0 | 7287 (67.1) | 9187 (64.7) |  |
| 1 | 720 ( 6.6) | 907 ( 6.4) |  |
| 2 | 710 ( 6.5) | 943 ( 6.6) |  |
| 3 | 2151 (19.8) | 3166 (22.3) |  |
| Suicide attempt (n,%) |  |  | 0.068 |
| 0 | 10627 (97.8) | 13944 (98.2) |  |
| 1 | 119 ( 1.1) | 121 ( 0.9) |  |
| 2 | 58 ( 0.5) | 57 ( 0.4) |  |
| 3 | 64 ( 0.6) | 81 ( 0.6) |  |
| Pressure score (n,%) |  |  | <0.001 |
| 0 | 9306 (85.6) | 10958 (77.2) |  |
| 1 | 758 ( 7.0) | 1432 (10.1) |  |
| 2 | 339 ( 3.1) | 650 ( 4.6) |  |
| 3 | 465 ( 4.3) | 1163 ( 8.2) |  |
| Multimorbidities |  |  |  |
| Hypertension (n,%) | 612 ( 5.6) | 788 ( 5.5) | 0.798 |
| Coronary artery disease (n,%) | 257 ( 2.4) | 457 ( 3.2) | <0.001 |
| Heart failure (n,%) | 356 ( 3.3) | 586 ( 4.1) | <0.001 |
| Chronic obstructive pulmonary disease (n,%) | 167 ( 1.5) | 307 ( 2.2) | <0.001 |
| Cerebral vascular disease (n,%) | 523 ( 4.8) | 710 ( 5.0) | 0.517 |
| Diabetes (n,%) | 280 ( 2.6) | 428 ( 3.0) | 0.042 |
| Cancers (n,%) | 31 ( 0.3) | 85 ( 0.6) | <0.001 |
| Chronic kidney disease (n,%) | 179 ( 1.6) | 342 ( 2.4) | <0.001 |
| Dementia (n,%) | 4094 (37.7) | 5483 (38.6) | 0.135 |
| Fracture (n,%) | 131 ( 1.2) | 168 ( 1.2) | 0.917 |
| Psychiatric diseases (n,%) | 2088 (19.2) | 3029 (21.3) | <0.001 |
| Physical ability score | 28.77 ±20.84 | 13.99 ±14.13 | <0.001 |
| Physical ability grade (n,%) |  |  | <0.001 |
| 1 | 628 ( 5.8) | 52 ( 0.4) |  |
| 2 | 2405 (22.1) | 289 ( 2.0) |  |
| 3 | 7835 (72.1) | 13862 (97.6) |  |
| Physical ability |  |  |  |
| Bowel control (n,%) |  |  | <0.001 |
| 0 | 3528 (32.5) | 8277 (58.3) |  |
| 5 | 4507 (41.5) | 4591 (32.3) |  |
| 10 | 2833 (26.1) | 1335 ( 9.4) |  |
| Bladder control (n,%) |  |  | <0.001 |
| 0 | 4262 (39.2) | 9437 (66.4) |  |
| 5 | 4379 (40.3) | 3838 (27.0) |  |
| 10 | 2227 (20.5) | 928 ( 6.5) |  |
| Grooming (n,%) |  |  | <0.001 |
| 5 | 1741 (16.0) | 817 ( 5.8) |  |
| Toilet use (n,%) |  |  | <0.001 |
| 0 | 6068 (55.8) | 11725 (82.6) |  |
| 5 | 4557 (41.9) | 2463 (17.3) |  |
| 10 | 243 ( 2.2) | 15 ( 0.1) |  |
| Feeding (n,%) |  |  | <0.001 |
| 0 | 4189 (38.5) | 9513 (67.0) |  |
| 5 | 5601 (51.5) | 4326 (30.5) |  |
| 10 | 1078 ( 9.9) | 364 ( 2.6) |  |
| Transferring (n,%) |  |  | <0.001 |
| 0 | 2379 (21.9) | 6288 (44.3) |  |
| 5 | 4001 (36.8) | 5434 (38.3) |  |
| 10 | 3957 (36.4) | 2397 (16.9) |  |
| 15 | 531 ( 4.9) | 84 ( 0.6) |  |
| Mobility on level surface (n,%) |  |  | <0.001 |
| 0 | 4935 (45.4) | 10162 (71.5) |  |
| 5 | 2985 (27.5) | 2906 (20.5) |  |
| 10 | 2557 (23.5) | 1056 ( 7.4) |  |
| 15 | 391 ( 3.6) | 79 ( 0.6) |  |
| Dressing (n,%) |  |  | <0.001 |
| 0 | 7000 (64.4) | 12174 (85.7) |  |
| 5 | 3498 (32.2) | 1968 (13.9) |  |
| 10 | 370 ( 3.4) | 62 ( 0.4) |  |
| Stairs (n,%) |  |  | <0.001 |
| 0 | 9165 (84.3) | 13675 (96.3) |  |
| 5 | 1627 (15.0) | 511 ( 3.6) |  |
| 10 | 76 ( 0.7) | 17 ( 0.1) |  |
| Bathing (n,%) |  |  | <0.001 |
| 5 | 125 ( 1.2) | 17 ( 0.1) |  |
| Cognitive ability score | 2.36 ±1.22 | 2.52 ±1.13 | <0.001 |
| Cognitive ability grade (n,%) |  |  | <0.001 |
| 0 | 376 ( 3.5) | 142 ( 1.0) |  |
| 1 | 2397 (22.0) | 2141 (15.1) |  |
| 2 | 6207 (57.0) | 9332 (65.7) |  |
| 3 | 1907 (17.5) | 2590 (18.2) |  |
| Perceptual ability grade (n,%) |  |  | <0.001 |
| 0 | 2642 (24.3) | 1349 ( 9.5) |  |
| 1 | 5492 (50.4) | 6398 (45.0) |  |
| 2 | 2512 (23.1) | 5648 (39.8) |  |
| 3 | 241 ( 2.2) | 810 ( 5.7) |  |
| Perceptual ability |  |  |  |
| Vision (n,%) |  |  | <0.001 |
| 0 | 1449 (13.3) | 931 ( 6.5) |  |
| 1 | 5355 (49.3) | 5520 (38.9) |  |
| 2 | 2789 (25.7) | 4756 (33.5) |  |
| 3 | 1275 (11.7) | 2997 (21.1) |  |
| Hearing (n,%) |  |  | <0.001 |
| 0 | 4547 (41.8) | 3312 (23.3) |  |
| 1 | 3410 (31.4) | 4400 (31.0) |  |
| 2 | 2486 (22.9) | 5359 (37.7) |  |
| 3 | 425 ( 3.9) | 1132 ( 8.0) |  |
| Communication ability (n,%) |  |  | <0.001 |
| 0 | 2665 (24.5) | 1386 ( 9.8) |  |
| 1 | 3463 (31.9) | 3522 (24.8) |  |
| 2 | 2607 (24.0) | 4075 (28.7) |  |
| 3 | 2133 (19.6) | 5220 (36.8) |  |

**Supplementary Table S5 Candidate predictors of mortality in older disabled population from Chengdu Long-term Care Insurance program**

| **Predictors** | | **Measurement scale** |
| --- | --- | --- |
| Time of Disability (month) | | Continuous |
| Rehabilitation therapy | | (1) Yes (2) No |
| Multiple drug use | | More than five drugs |
| Socio-demographics | Sex | (1) Male (2) Female |
|  | Age | Continuous |
|  | BMI (kg/m2) | 0: <18.5 |
|  |  | 1:18.5-24.5 |
|  |  | 2: > 24.5 |
|  | Education level | (1) Illiteracy or primary school (2) Junior high school or above |
|  | Marital status | (1) Married (2) Divorced/Widowed/Unmarried |
|  | Living status | (1) Lived in professional nursing institution (2) Lived in household |
|  | Smoking | (1) Yes (2) No |
|  | Drinking | (1) Yes (2) No |
| Clinical signs | Distolic blood pressure (mmHg) | Continuous |
|  | Systolic blood pressure (mmHg) | Continuous |
|  | Heart rate | (1) Normal (2) Abnormal |
|  | Respiratory rate | (1) Normal (2) Abnormal |
|  | Consciousness | (1) Conscious (2) Drowsiness (3) Lethargy (4) Coma |
| Injury events | Scalds | (1) Yes (2) No |
|  | Get lost | (1) Yes (2) No |
|  | Fall during walking | (1) Yes (2) No |
|  | Fall out of bed | (1) Yes (2) No |
|  | Choke on food | (1) Yes (2) No |
|  | Suicide attempt | (1) Yes (2) No |
|  | Pressure sores | (1) Yes (2) No |
| Comorbidities | Hypertension | (1) Yes (2) No |
|  | Coronary artery disease | (1) Yes (2) No |
|  | Heart failure | (1) Yes (2) No |
|  | Chronic obstructive pulmonary disease | (1) Yes (2) No |
|  | Cerebral vascular disease | (1) Yes (2) No |
|  | Diabetes | (1) Yes (2) No |
|  | Cancers | (1) Yes (2) No |
|  | Chronic kidney disease | (1) Yes (2) No |
|  | Dementia | (1) Yes (2) No |
|  | Fracture | (1) Yes (2) No |
|  | Psychiatric diseases | (1) Yes (2) No |
| Physical ability score | | Continuous |
| Physical ability grade | | (0) Robust 100 |
|  |  | (1) Mild impairment 65-95 |
|  |  | (2) Moderate impairment 45-60 |
|  |  | (3) Serious impairment ≤40 |
| Physical ability | Bowel control | 0= incontinent (or needs to be given enemas) |
|  |  | 5 = occasional accident |
|  |  | 10 = continent |
|  | Bladder control | 0 = incontinent, or catheterized and unable to manage alone |
|  |  | 5 = occasional accident |
|  |  | 10 = continent |
|  | Grooming | 0 = needs to help with personal care |
|  |  | 5 = independent face/hair/teeth/shaving (implements provided) |
|  | Toilet use | 0 = dependent |
|  |  | 5 = needs some help, but can do something alone |
|  |  | 10 = independent (on and off, dressing, wiping) |
|  | Feeding | 0 = unable |
|  |  | 5 = needs help cutting, spreading butter, etc., or requires modified diet |
|  |  | 10 = independent |
|  | Transferring | 0 = unable, no sitting balance |
|  |  | 1 = major help (one or two people, physical), can sit |
|  |  | 2 = minor help (verbal or physical) |
|  |  | 3 = independent |
|  | Mobility on level surface | 0 = immobile or < 50 yards |
|  |  | 5 = wheelchair independent, including corners, >50 yards |
|  |  | 10 = walks with help of one person (verbal or physical) > 50 yards |
|  |  | 15 = independent (but may use any aid; for example, stick) > 50 yards |
|  | Dressing | 0 = dependent |
|  |  | 5 = needs help but can do about half unaided |
|  |  | 10 = independent (including buttons, zips, laces, etc.) |
|  | Stairs | 0 = unable |
|  |  | 5 = needs help (verbal, physical, carrying aid) |
|  |  | 10 = independent |
|  | Bathing | 0 = dependent |
|  |  | 5 = independent (or in shower) |
| Cognitive ability grade | | (0) Robust 0 |
|  |  | (1) Mild impairment 1-2 |
|  |  | (2) Moderate impairment 3-5 |
|  |  | (3) Serious impairment 6 |
| Cognitive ability | Memory | 2= unable to tell the birth date or birthplace, or what is doing before the assessment |
|  |  | 1= partially able to tell the birth date, birthplace, and what is doing before the assessment |
|  |  | 0= able to tell the birth date, birthplace, and what is doing before the assessment |
|  | Orientation | 2= unable to tell the location or time, and unable to distinguish people |
|  |  | 1= partially able to tell the location and time, and distinguish people |
|  |  | 0= able to tell the location and time, and distinguish people |
|  | Judgment and execution | 2= unable to follow instructions or count |
|  |  | 1= partially able to follow instructions and count |
|  |  | 0= able to follow instructions and count |
| Perceptual ability grade | | (0) Robust 0 |
|  |  | (1) Mild impairment 1-4 |
|  |  | (2) Moderate impairment 5-8 |
|  |  | (3) Serious impairment 9 |
| Perceptual ability | Vision | 3= only light perception |
|  |  | 2= can identify large object |
|  |  | 1= can see large font clearly |
|  |  | 0= can see standard font clearly |
|  | Hearing | 3= completely inaudible |
|  |  | 2= partially heard when speaking loudly |
|  |  | 1= inaudible when Speaking from a distance of more than 2 meters |
|  |  | 0= converse normally |
|  | Communication ability | 3= unable to express their needs or understand others |
|  |  | 2= express their needs and understand other hardly, frequently repetition or simplifying expression is required |
|  |  | 1= able to express their needs and understand others barely |
|  |  | 0= communicate normally |

**Supplementary Figure S1. Thirty clinical features identified by LASSO model**


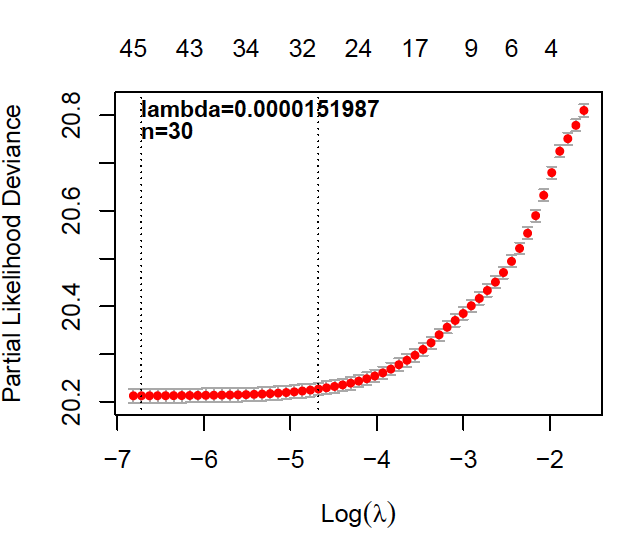


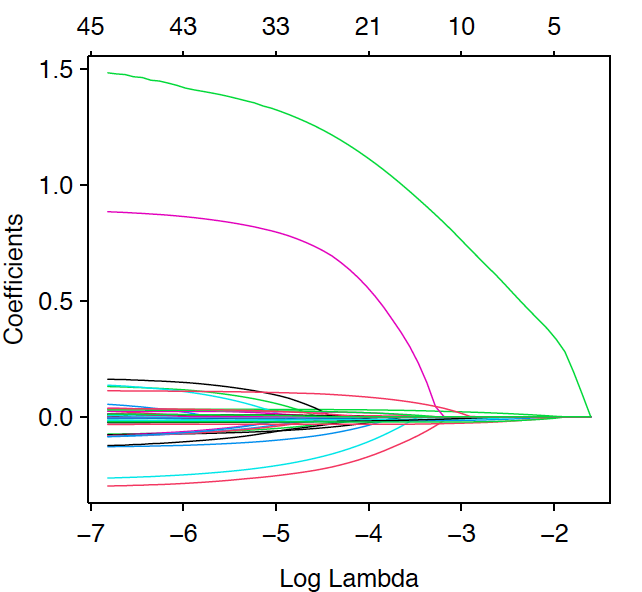


**Supplementary Figure S2. Precision- Recall curve of deep learning model and Cox model**


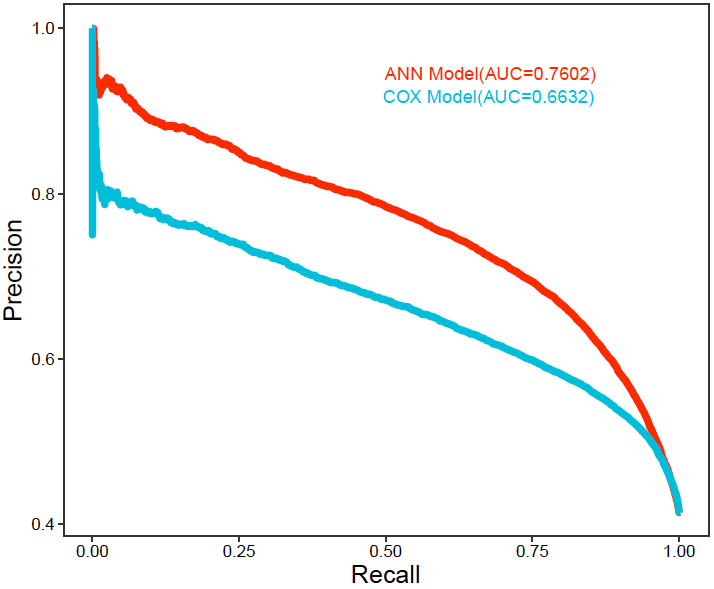


**Supplementary Figure S3 Kaplan-Meier curves of the low-, medium-, and high-risk groups, stratified by traditional Cox regression**

**
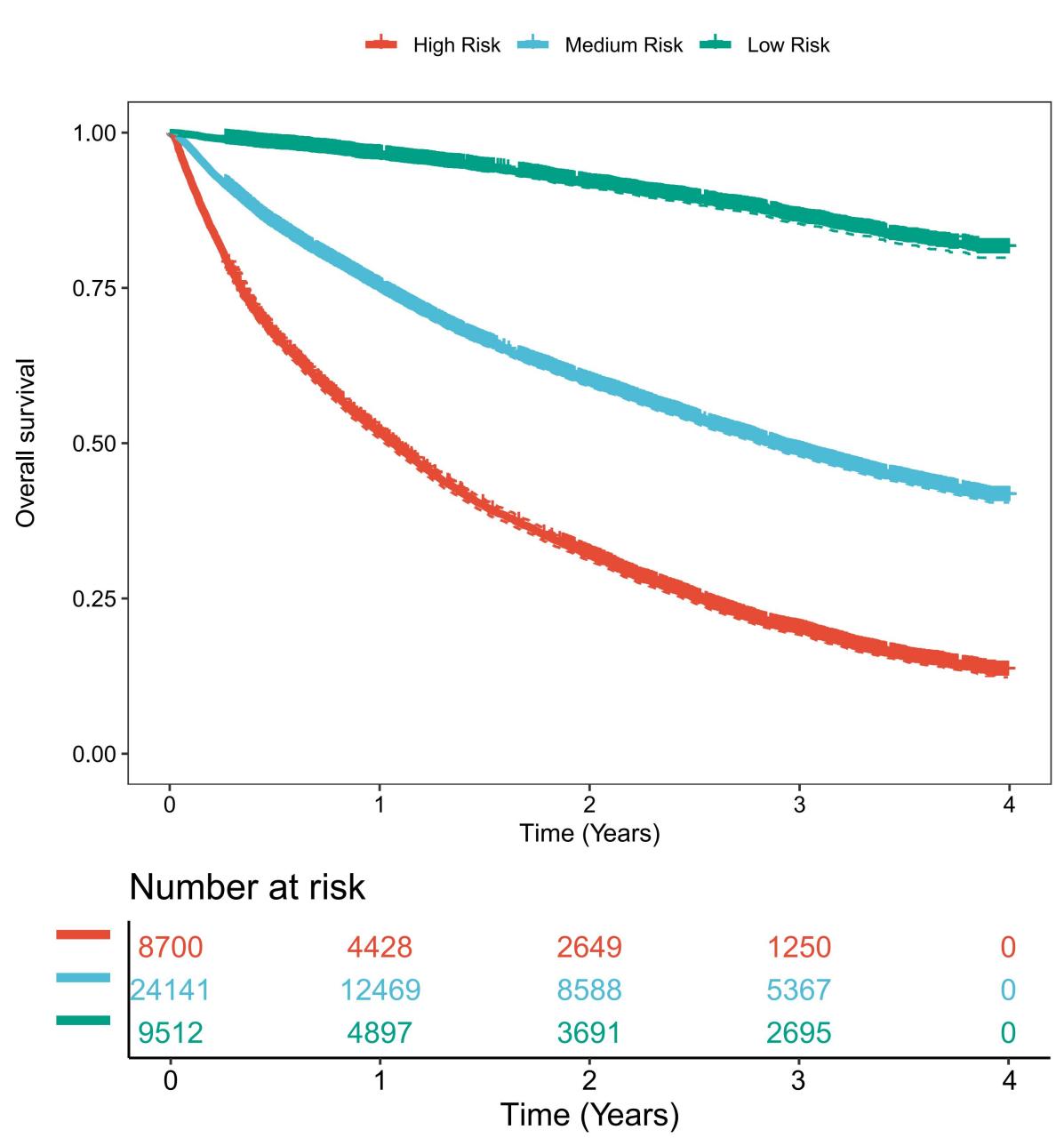
**

**Supplementary Figure S4. Flowchart of LTCI application process**


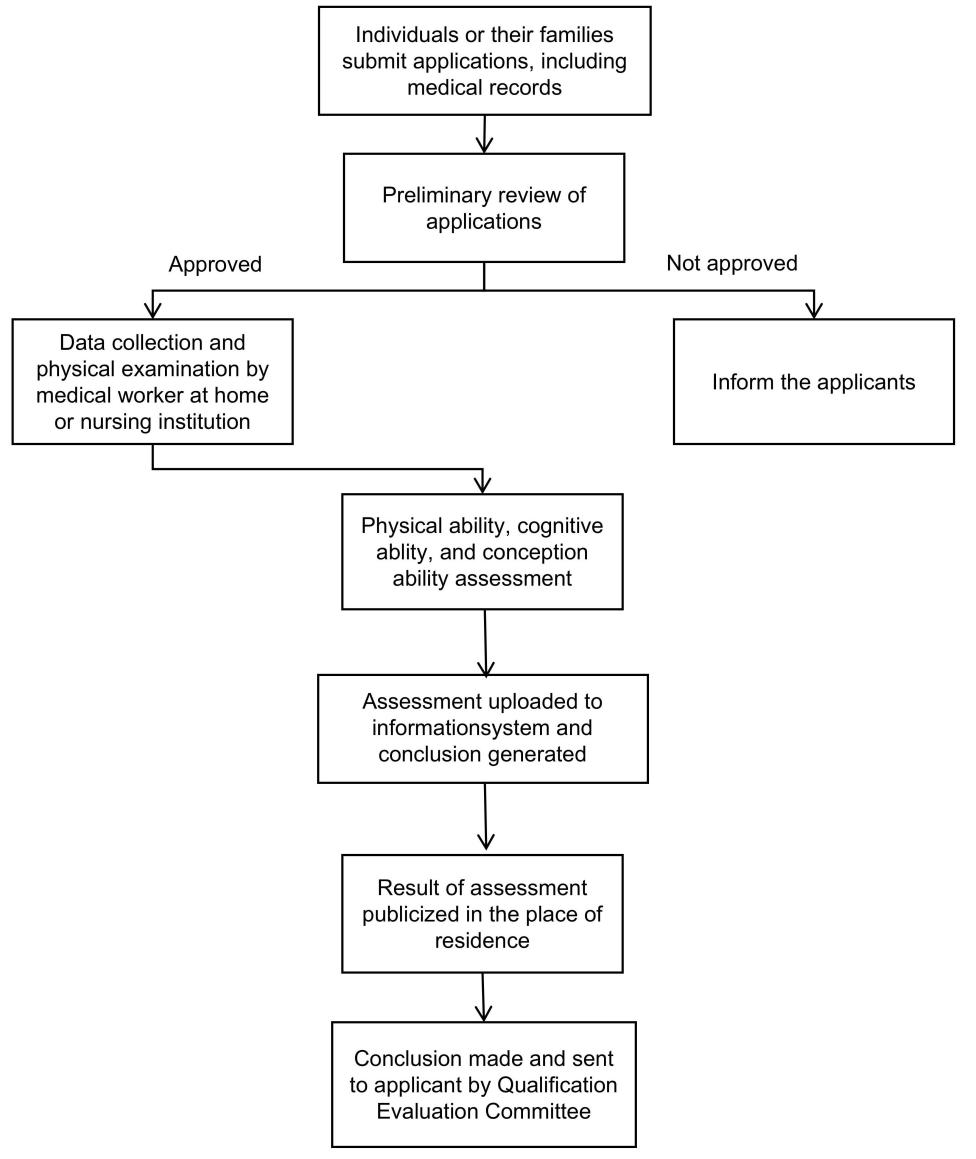


**Supplementary Figure S5. Schematic diagram of optimal Artificial Neural Network model**


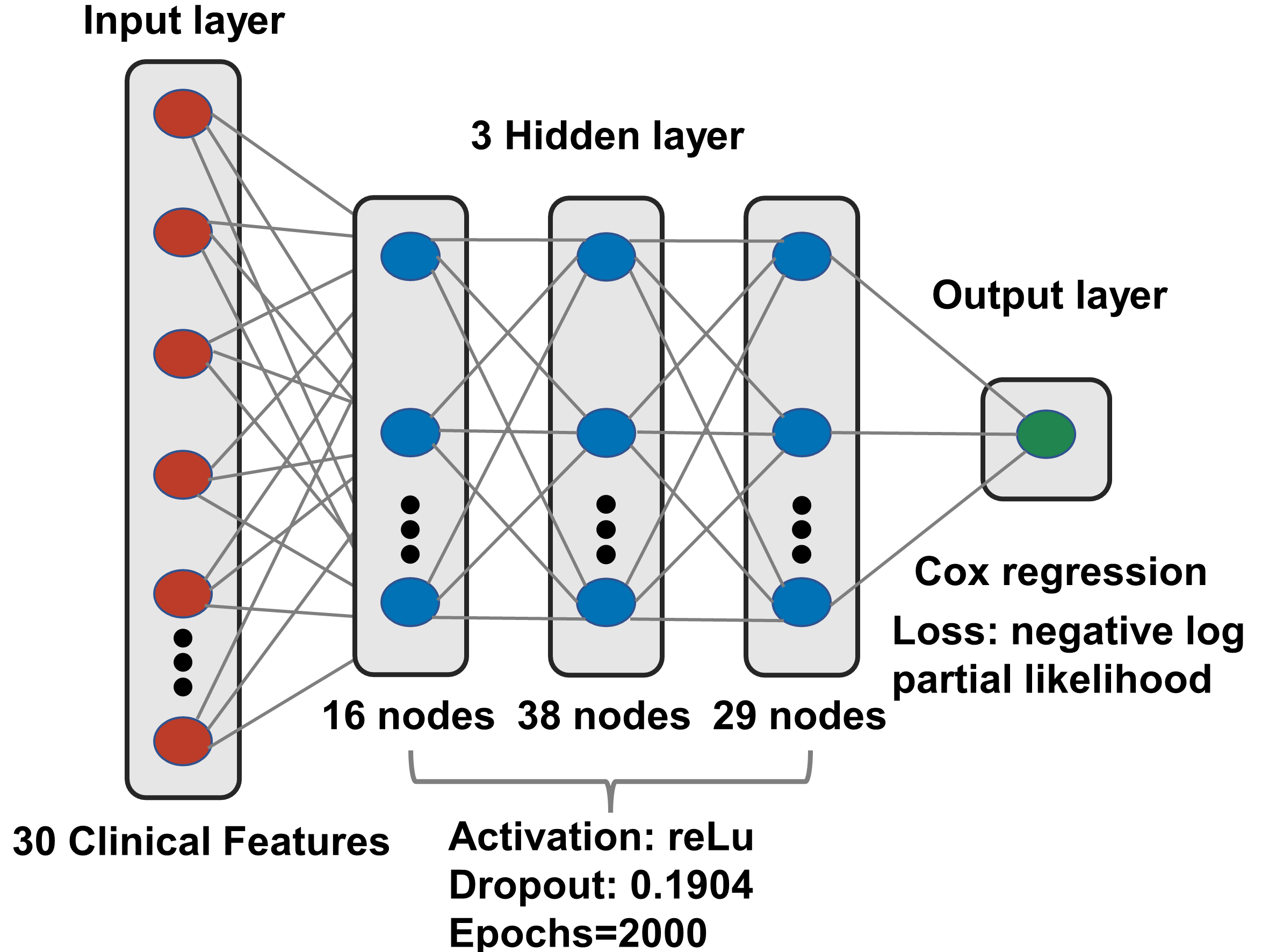

Supplement: Supplementary file 2 — Supplementary Information 2. [file 41598_2024_65601_MOESM2_ESM.docx]
